# Mutational landscape of mucinous ovarian carcinoma and its neoplastic precursors

**DOI:** 10.1186/s13073-015-0210-y

**Published:** 2015-08-07

**Authors:** Georgina L. Ryland, Sally M. Hunter, Maria A. Doyle, Franco Caramia, Jason Li, Simone M. Rowley, Michael Christie, Prue E. Allan, Andrew N. Stephens, David D L Bowtell, Australian Ovarian Cancer Study Group, Ian G. Campbell, Kylie L. Gorringe

**Affiliations:** Cancer Genetics Laboratory, Peter MacCallum Cancer Centre, East Melbourne, Victoria Australia; Bioinformatics Core Facility, Peter MacCallum Cancer Centre, East Melbourne, Victoria Australia; Department of Anatomical Pathology, Royal Melbourne Hospital, Parkville, Victoria Australia; Department of Pathology, Peter MacCallum Cancer Centre, East Melbourne, Victoria Australia; Centre for Cancer Research, MIMR-PHI Institute of Medical Research, Clayton, Victoria Australia; Department of Molecular and Translational Sciences, Monash University, Clayton, Victoria Australia; Epworth Research Institute, Epworth HealthCare, Richmond, Victoria Australia; Cancer Genetics and Genomics Laboratory, Peter MacCallum Cancer Centre, East Melbourne, Victoria Australia; Sir Peter MacCallum Department of Oncology, University of Melbourne, Parkville, Victoria Australia; Department of Biochemistry and Molecular Biology, University of Melbourne, Parkville, Victoria Australia; Department of Pathology, University of Melbourne, Parkville, Victoria Australia

## Abstract

**Background:**

Mucinous ovarian tumors are an unusual group of rare neoplasms with an apparently clear progression from benign to borderline to carcinoma, yet with a controversial cell of origin in the ovarian surface epithelium. They are thought to be molecularly distinct from other ovarian tumors but there have been no exome-level sequencing studies performed to date.

**Methods:**

To understand the genetic etiology of mucinous ovarian tumors and assess the presence of novel therapeutic targets or pathways, we undertook exome sequencing of 24 tumors encompassing benign (5), borderline (8) and carcinoma (11) histologies and also assessed a validation cohort of 58 tumors for specific gene regions including exons 4–9 of *TP53*.

**Results:**

The predominant mutational signature was of C>T transitions in a NpCpG context, indicative of deamination of methyl-cytosines. As well as mutations in known drivers (*KRAS*, *BRAF* and *CDKN2A*), we identified a high percentage of carcinomas with *TP53* mutations (52 %), and recurrent mutations in *RNF43*, *ELF3*, *GNAS*, *ERBB3* and *KLF5*.

**Conclusions:**

The diversity of mutational targets suggests multiple routes to tumorigenesis in this heterogeneous group of tumors that is generally distinct from other ovarian subtypes.

**Electronic supplementary material:**

The online version of this article (doi:10.1186/s13073-015-0210-y) contains supplementary material, which is available to authorized users.

## Background

Epithelial ovarian tumors have historically been treated as a homogenous group in the clinic, despite clear histopathological and molecular data showing that distinct subgroups exist: serous, endometrioid, clear cell and mucinous. High-grade serous and low-grade serous tumors comprise distinct groups, while endometrioid and clear-cell histologies are different again from serous but with some overlapping genetic events. It is now clear that these molecular distinctions reflect differences in site of origin, with high-grade serous tumors now thought to arise from the fallopian tube fimbriae, low-grade serous tumors from the ovarian epithelium, and clear cell and endometrioid tumors arising from endometriosis, which itself is derived from the endometrium. However, the origin of the mucinous group remains controversial. Many mucinous ovarian tumors (MOTs) formerly classified as primary are now recognized to have been mis-diagnosed metastases from predominantly gastrointestinal or endocervical sites. However, some mucinous tumors do appear to be ovarian primaries, particularly benign and borderline tumors, which generally have a good prognosis not consistent with a metastatic tumor. Carcinomas associated with benign and borderline elements and/or with an early-stage, unilateral presentation are also thought to be primary ovarian in origin.

Our understanding of the genomic landscape of MOTs is limited. Older reports are likely to include a high proportion of metastatic mucinous tumors, and the rarity of true primary mucinous tumors results in limited investigations. Nonetheless, we and others have shown that mucinous tumors have a high proportion of mutations in RAS pathway genes and aberration of *CDKN2A* (p16) [1, 2]. Beyond these common drivers, little is known, and the predominantly stable genomic copy number profiles we have observed in this tumor type [3] suggest that somatic point mutations are likely to be more relevant. In this study, we have undertaken exome sequencing of a large cohort of MOTs, and have further investigated lead candidates in a validation cohort of 58 cases.

## Methods

### Specimens

Fresh-frozen MOTs were accessed from bio-banked specimens collected and cryopreserved at the time of surgical resection for a primary ovarian tumor, prior to chemotherapy administration. Samples comprised 22 benign cystadenomas, 29 tumors of low malignant potential (herein referred to as borderline tumors) and 31 carcinomas [4, 5]. Hospitals contributing samples between 1993 and 2011 included those in the south of England, UK [5], and in Australia (Southern Health and the Australian Ovarian Cancer Study [AOCS] [4]). Blood samples used for germline DNA extraction were also collected prior to surgery. Thorough histological classification was based on the entire specimen at time of diagnosis, although all cases underwent retrospective pathological review using information obtained from the pathology report and histological assessment according to established criteria [6] in order to exclude likely metastases. Cases were also excluded if there was insufficient tumor epithelium for nucleic acid extraction. Carcinoma grade was derived from the diagnostic pathology report because there were insufficient cases with archival specimens available for re-review. Clinicopathological data are provided in Additional file 1: Table S1.

This study was performed in accordance with the ethical standards of the Peter MacCallum Cancer Centre Human Research Ethics Committee (Approvals 09/29 and 01/38) and all participants provided written informed consent for tissue collection. This study conforms to the Declaration of Helsinki.

### DNA extraction

Tumor genomic DNA was isolated by needle microdissection of areas with greater than 80 % neoplastic cellularity from consecutive 10 μm hematoxylin and eosin (H&E)-stained tumor sections and extracted using the DNeasy Blood and Tissue Kit (Qiagen, Valencia, CA, USA) as per the recommended protocol. Matched germline DNA was extracted from whole blood (19 cases) or paired uninvolved ovarian stroma (5 cases). Whole genome amplification (WGA) was performed on 20–50 ng of tumor and germline DNA using the Repli-G Phi-mediated amplification system (Qiagen) and the product was used to confirm mutations detected by exome sequencing and to perform candidate gene mutation analysis.

### Whole-exome library construction and sequencing

Libraries were constructed from 500 ng of unamplified tumor or germline DNA following the Illumina TruSeq DNA Sample Preparation procedure (Illumina, San Diego, CA, USA), followed by exome capture using the NimbleGen SeqCap EZ Human Exome Library v1 or v2 capture kit (Roche NimbleGen, Heidelberg, Germany). Each resulting paired-end library was sequenced on one-third of an Illumina HiSeq2000 lane using 75 bp or 100 bp reads. Library preparation and detailed summary statistics for all samples are listed in Additional file 1: Table S2.

### Somatic mutation analysis

Purity filtered paired-end reads were quality checked with FastQC (v0.10.1) and trimmed for low quality bases and adaptor if necessary using Cutadapt (v1.1). Reads were then aligned to the human genome (GRCh37/hg19) using BWA-MEM (v0.7.7-r441). Duplicates were marked using Picard (v1.77) followed by local insertion-deletion (indel) re-alignment and base quality score recalibration using GATK (v2.7-2-g6bda569). Somatic single nucleotide variants (SNVs) and indels were called using the following algorithms with the matched germline data used as reference: MuTect (v2.7-1-g42d771f), JointSNVMix (v0.8-b2) and Somatic Sniper (1.0.2.2-1-g8ee3999) (SNVs only); SomaticIndelDetector (v1.0.4905) (indels only); and VarScan (v2.3.4) (both SNVs and indels). In addition, Pindel (v0.2.5a3) and GATK Unified Genotyper (v2.7-2-g6bda569) were used to call SNVs and indels separately in the tumor and germline samples.

Initial variant predictions were filtered to require that (1) SNVs were called by ≥2 of MuTect, JointSNVMix, Somatic Sniper, VarScan or Unified Genotyper, (2) indels were called by any of SomaticIndelDetector, VarScan, Pindel or Unified Genotyper, (3) the variant was present in ≥10 reads in the tumor (Pindel) or ≥2 reads in the tumor (all other callers), (4) the mutant allele frequency was ≤5 % in the matched germline sample, and (5) the mutant allele fraction was at least 10 % higher in the tumor than in the matched germline sample for indels called by Pindel and Unified Genotyper. Finally, any remaining germline single nucleotide polymorphisms (SNPs) or common sequence artifacts were eliminated by requiring that the variant allele was not observed in more than two of the other germline samples from this cohort or more than two (of 147) in-house germline exome sequences [7], and had an Exome Variant Server (ESP6500 SI-v2) minor allele frequency of ≤5 %.

Predicted somatic mutations were annotated with Ensembl v73 information and those with impact predictions overlapping coding regions and splice sites (±2 bp) were considered for further analysis. Due to restrictions of our ethics approval, we are not able to provide BAM files; however, all variants are available in Additional file 1: Table S3. All coding mutations were manually reviewed by examination of BAM files using the Integrative Genomics Viewer.

### Mutation confirmation by nucleotide sequencing

Selected somatic mutations were independently assessed by polymerase chain reaction (PCR) and Sanger sequencing of the tumor DNA as described previously [8]. Somatic status was confirmed by also resequencing the corresponding germline sample.

Thirty-two known somatic mutations in *KRAS*, *BRAF*, *TP53* and *CDKN2A* that had been independently validated in other studies of this cohort by Sanger sequencing were used to assess the sensitivity of somatic mutation calling, with 96.9 % known somatic variants successfully identified (27/27 SNVs and 4/5 indels). Failure to identify a known 36 bp complex indel in *CDKN2A* was complicated by low read depth owing to high GC content for this gene; this variant was included in subsequent analyses. The confirmation rate of novel variants by Sanger sequencing was 93.6 % (208/223 SNVs and 25/26 indels).

### Significantly mutated gene prediction

The MuSiC algorithm (v0.4) was applied using default parameters to identify genes significantly enriched for mutations, given sequence type, context and estimated background rate [9]. Genes mutated in two or more samples with a *P*-value ≤0.05 at a false discovery rate of ≤0.1 in any of the three tests were considered significant. Furthermore, OncodriveFM (accessed through the online IntOGen platform at [10]) was used to identify genes with significant bias towards the accumulation of functional mutations (*P*-value ≤0.05 and *q*-value ≤0.1) [11, 12].

### Mutation analysis of *ELF3*, *ERBB3*, *GNAS*, *TP53*, *RAS-RAF* and *CDKN2A* by Sanger sequencing

The complete coding exons of *ELF3* (exons 2–9) and *ERBB3* (exons 1–28) were assessed by direct Sanger sequencing using the primers listed in Additional file 1: Table S4. To assess the *ELF3* c.1001 + 1_1001 + 2insGG mutation on mRNA splicing, cDNA amplification and direct sequencing were performed using primers listed in Additional file 1: Table S4. Targeted Sanger sequencing of mutation hotspots in *TP53* (exons 4–9), *BRAF* (codon 600), *KRAS*/*HRAS*/*NRAS* (codons 12, 13 and 61) and the coding region of *CDKN2A* (exons 1–3) were sequenced using primers previously described [2]. Somatic mutations identified in these genes have previously been published for a subset of the benign and borderline mucinous tumors [2].

### Mutation analysis of *GNAS* and *KLF5* by high-resolution melt analysis

High-resolution melt analysis was used to screen for mutations at hotspot codon 201 of *GNAS* and in the coding exons of *KLF5* (exons 1–4). For this, 15 ng WGA tumor DNA was amplified in duplicate using the primers listed in Additional file 1: Table S4, followed by melt analysis on the LightCycler 480 Instrument using Gene Scanning Software (Roche). Samples with variant melt curves in duplicate PCRs were independently amplified using the same primers (*KLF5*) or an independent primer set (*GNAS*) and Sanger sequenced to confirm sequence variations.

### Analysis of *CDKN2A* and *HER2*

HER2 status was ascertained based on the detection of high-level gene amplification by high-density genome-wide SNP arrays (Affymetrix, Santa Clara, CA, USA) (35 cases) [2, 3], SNP array data plus immunohistochemistry (IHC) (23 cases), or by IHC alone (18 cases). For 16 cases, HER2 IHC was evaluated on 4 μM formalin-fixed paraffin-embedded whole sections using anti-HER2 antibody clone 4B5 (Ventana Medical Systems, Tuscon, AZ, USA). Staining was scored visually according to standard guidelines [13]; briefly, an IHC score of 3+ (strong uniform membrane staining of >30 % tumor cells) was categorized as HER2 positive; equivocal cases (score of 2+, strong complete membrane staining in <30 % tumor cells or weak to moderate heterogeneous staining in >10 % tumor cells) were only considered HER2 positive if accompanied by array-based copy number amplification of the *ERBB2* locus. An equivocal score without amplification confirmation, and tumors that scored 0 or 1 (no staining or weak incomplete membrane staining in any proportion of tumor cells), were considered negative. For the remaining 25 cases, the HER2 IHC score was derived from Anglesio et al. [1], who used comparable classification guidelines.

## Results and discussion

### Somatic mutation frequency and spectra

**Fig. 1:**
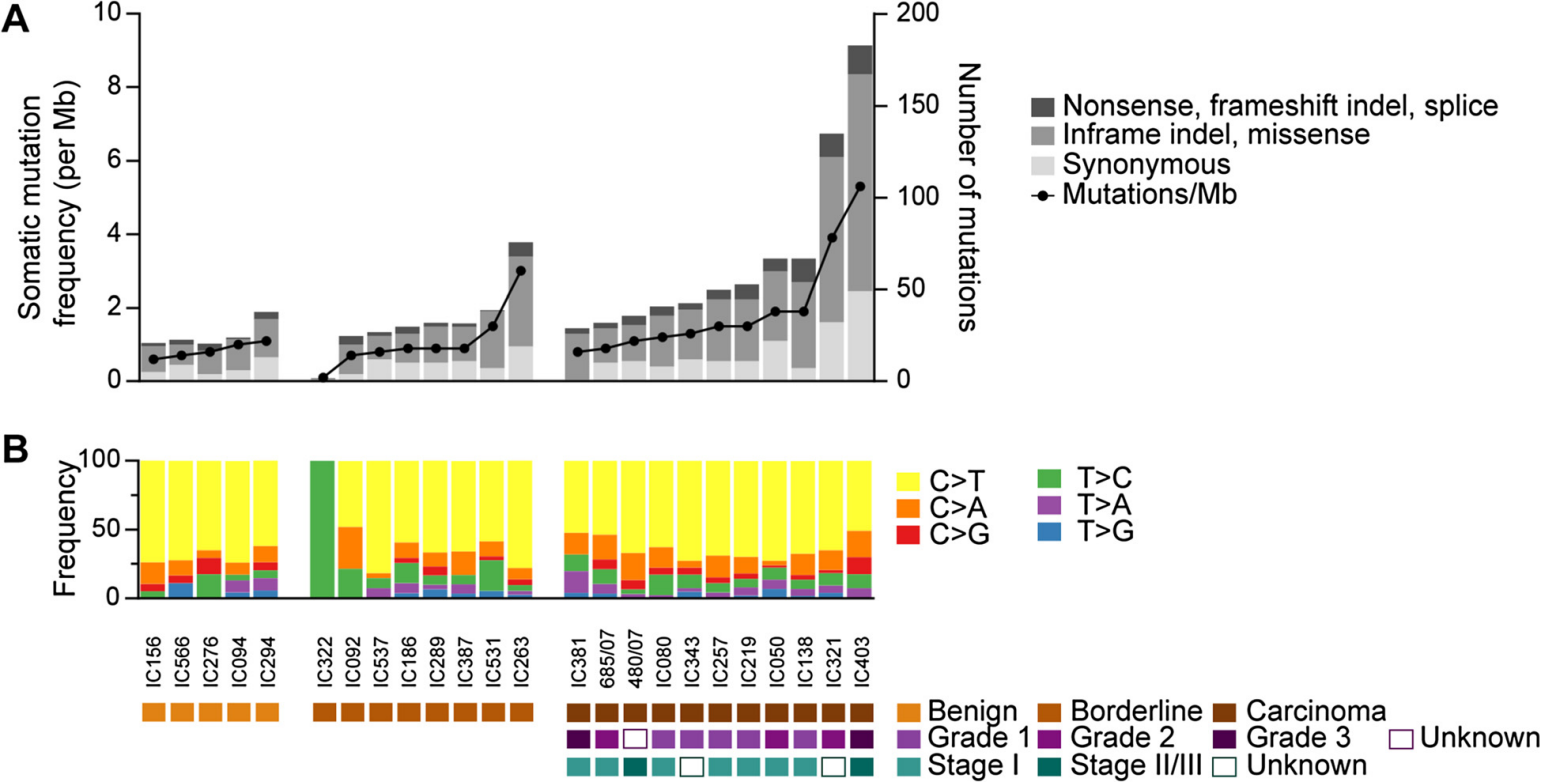
Mutational landscape of MOTs identified by exome sequencing. Samples are grouped according to pathological classification and ordered from lowest to highest mutation frequency. **a** Somatic mutation frequency (left Y-axis) and number of coding mutations by consequence (right Y-axis). **b** Relative frequency of somatic mutations according to base substitution type. Substitutions were categorized by the six possible base-pair changes

To profile the somatic mutation spectrum of mucinous tumors of the ovary, we performed whole exome sequencing on 24 tumors including 5 benign cystadenomas, 8 borderline tumors and 11 carcinomas (Table 1, Additional file 1: Table S1). A mean coverage depth of 144× was achieved in both neoplastic and non-cancerous specimens (range 53-fold to 224-fold) and 91 % of the bases were covered by at least 20 uniquely mapping reads (Additional file 1: Table S2). Using stringent criteria, 1126 somatic coding and essential splice site mutations were identified (1031 SNVs and 95 indels), of which 841 were predicted to alter protein sequence (Additional file 1: Table S3). These included 44 (5.2 %) nonsense, 60 (7.1 %) frameshift indel, 16 (1.9 %) splice site, 27 (3.2 %) inframe indel and 694 (82.5 %) missense mutations. Benign and borderline tumors had on average 25.4 (range 21–38) and 32.9 (range 2–76) coding mutations per tumor, equating to a frequency of 0.8 mutations/Mb and 0.9 mutations/Mb respectively. Although variable, this mutation burden did not differ between benign and borderline tumors but was significantly lower when compared to the carcinomas (average of 66.9 mutations per sample and 1.5 mutations/Mb) (*P* = 0.008 vs. benign and *P* = 0.047 vs. borderline) attributed mostly to an accumulation of missense mutations in the carcinomas (Fig. 1a, Additional file 1: Table S5). There were no hyper-mutated cases (defined as >10 mutations/Mb) indicative of a mutator phenotype such as mismatch repair deficiency. Relative to other cancer types, MOTs showed a similar somatic mutation density to breast, serous ovarian and pancreatic cancers, but a lower density than colorectal and stomach tumors [14, 15].

**Table 1:** Cohort summary

Clinical feature	Discovery (n = 24)	Validation (n = 58)
Type:		
Benign	5	17
Borderline	8	21
Carcinoma	11	20
Grade (carcinoma)		
1	5	6
2	3	10
3	2	4
Not known	1	
Stage (carcinoma)		
1	7	16
2	1	1
3	1	1
Not known	2	2
Age (average ± standard deviation)		
Benign	49.8 ±5.9	59.5 ±11.3
Borderline	55.4 ±15.8	53.6 ±16.0
Carcinoma	62.3 ±11.0	53.8 ±11.1
Laterality		
Unilateral	21	54
Bilateral	2	4
Not known	1	
Size		
<10 cm	2	1
≥10 cm	21	57
Not known	1	0

The mutation spectrum was dominated by C>T transitions, comprising 63.9 % of somatic substitutions, and this was common to all three tumor subtypes (Fig. 1b). Mutations in this context demonstrated a marked preference for NpCpG trinucleotides (Additional file 2, Figure S1a), the optimum motif for spontaneous 5-methylcytosine deamination [16]. An equivalent signature is frequently seen in other epithelial tumors of the gastrointestinal tract, but is different to that observed in other cancers of the female reproductive system including high-grade serous ovarian carcinoma (Additional file 2: Figure S1b). Taken together these findings are consistent with MOTs having a shared lineage distinct from that of other ovarian epithelial tumors.

### Profile of mutated genes in mucinous ovarian tumors

**Fig. 2:**
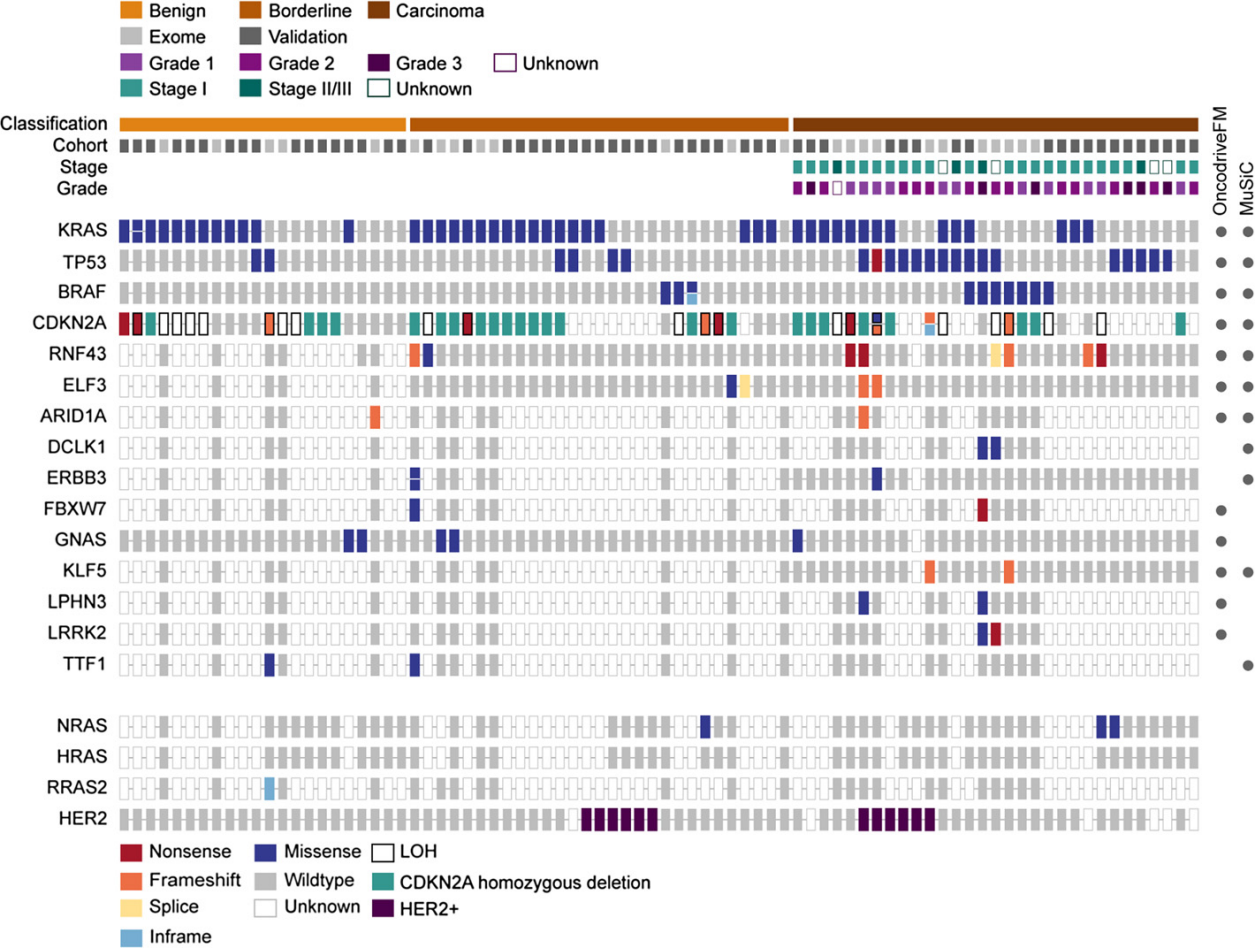
Candidate driver genes in MOTs. Significantly mutated genes identified by OncodriveFM and MuSiC analyses are arranged vertically by their frequency of mutated samples in the whole exome sequencing data. *Color* indicates mutation consequence. Selected genes were also investigated in a validation cohort of mucinous tumors. Each *column* denotes an individual tumor (ordered as listed in Additional file 1: Table S1), which have been arranged to emphasize mutational groups. Genomic aberrations in other MAPK pathway genes were also screened for mutations. *LOH* loss of heterozygosity

Protein-altering mutations were detected in 761 genes, of which 42 were mutated in two or more of the 24 tumors. Among the most frequently mutated were known mucinous ovarian cancer genes *KRAS*, *BRAF* and *CDKN2A* (Table 2, Fig. 2). Interestingly, *TP53* was the second most frequently mutated gene, with seven mutations identified. Eight genes were significantly mutated based on a statistically significant accumulation of mutations by both MuSiC [9] and OncodriveFM [11] (Table 2). Other genes predicted by one algorithm were also notable; for example, *ERBB3* (MuSiC) and *GNAS* and *FBXW7* (OncodriveFM) (Table 2). Based on these predictions and observation in other cancer types, five novel candidate drivers not previously reported in MOTs were selected for validation in an independent cohort: *TP53* (7/24), *ELF3* (3/24), *ERBB3* (2/24), *GNAS* (2/24) and *KLF5* (2/24) (Table 2, Fig. 2, Additional file 1: Table S6). Our validation study of the tumor suppressor gene *RNF43* has been published previously [8]. In addition, known cancer genes for this ovarian subtype were evaluated in parallel to assess their relationship with new drivers including HER2 (by immunohistochemistry and copy number analysis) and mutations in *KRAS*, *BRAF*, *CDKN2A* and other RAS pathway members *NRAS* and *HRAS*.

**Table 2:** Candidate driver genes with significantly recurrent somatic mutations in mucinous ovarian tumors

	Exome cohort			SMG prediction				Validation cohort			Overall
Gene	Mutated samples	Nonsense, frameshift indel, splice	Inframe indel, missense	OncodriveFM	MuSiC *q*-value			Mutated samples	Nonsense, frameshift indel, splice	Inframe indel, missense	Mutated samples
				*q*-value	FCPT	LRT	CT				
*KRAS*	12	0	12	1.34 × 10^−13^	0	0	0	32	0	33	44/82
*TP53*	7	1	6	3.66 × 10^−11^	2.86 × 10^−7^	1.41 × 10^−9^	1.17 × 10^−12^	15	0	15	22/82
*BRAF*	6	0	6	2.45 × 10^−8^	7.77 × 10^−7^	0	6.18 × 10^−12^	4	0	5	10/82
*CDKN2A*	5	5	2	0.0043	1.20 × 10^−10^	0	7.93 × 10^−17^	5	5	0	10/63
*RNF43*	5	5	0	4.65 × 10^−6^	–	0.0009	0.0004	3	2	1	8/65^b^
*ELF3*	3	2	1	0.0079	–	0.0003	0.0004	1	1	0	4/65
*ARID1A*	2	2	0	0.0164	–	0.0933	–	–	–	–	2/24
*DCLK1*	2	0	2	–	–	0.0569	–	–	–	–	2/24
*ERBB3*	2	0	3	–	–	0.0014	0.0374	0	0	0	2/43
*FBXW7*	2	1	1	0.0207	–	–	–	–	–	–	2/24
*GNAS* ^a^	2	0	2	9.05 × 10^−8^	–	–	–	3	0	3	5/81
*KLF5*	2	2	0	0.0164	–	0.0056	0.0536	0	0	0	2/43
*LPHN3*	2	0	2	0.0493	–	–	–	–	–	–	2/24
*LRRK2*	2	1	1	0.0997	–	–	–	–	–	–	2/24
*TTF1*	2	0	2	–	–	0.0569	–	–	–	–	2/24

All non-synonymous mutations in listed genes were validated by Sanger sequencing

*CT* convolution test, *FCPT* Fisher’s combined *P*-value test, *LRT* likelihood ratio test, *SMG* significantly mutated gene

^a^Only mutations involving the hotspot codon 201 are reported

^b^Includes samples from Ryland et al. [8] plus 16 additional samples

### Prevalence of mutations in known mucinous ovarian cancer genes

We and others have previously described the importance of the RAS pathway and p16 in MOTs [1–3]. Here we extended this analysis and found 56 cases with mutations in *KRAS*, *BRAF* and *NRAS* (68.3 %). *BRAF* mutations were significantly more prevalent in the carcinomas (7/31, 22.6 %) than in the borderline (3/29, 10.3 %) or benign tumors (0/22, *P* = 0.036 Fisher’s exact test), suggesting an association with a more aggressive phenotype. An alternative mechanism for activation of the MAPK pathway was identified through mutation of the *ras*-like gene *RRAS2* in one benign tumor that was *KRAS*/*BRAF* wild type (Fig. 2). We previously reported *RRAS2* gene amplification in this sample [2]; consistent with this, the Sanger sequencing validation confirmed homozygous amplification of the mutant allele (Additional file 2: Figure S2). This 9 bp duplication, resulting in reiteration of Gly-Gly-Gly (codons 22–24), occurs in the region of *RRAS2* that is complementary to codons 11–13 within the G1 phosphate-binding loop of conventional ras proteins (P-loop, amino acids 10–17). Interestingly, rare reports of comparable events appear in the literature. Huang et al*.* [17] described a three amino acid *RRAS2* duplication (Gly24_26dup) in the human uterine leiomyosarcoma cell line ST-UT-1; this mutation resulted in enhanced GTP-binding and conferred transforming activity *in vitro*. Similarly, in *KRAS*, 9 bp and 12 bp tandem repeats of codons 10–12 and 10–13 respectively were identified as an alternative mechanism for *KRAS* oncogenic activation in 2 of 18 chemically induced rat renal mesenchymal tumors [18]. Triple residue insertions in the P-loop of *HRAS* also demonstrated increased preference for GTP-binding and increased interactions with downstream Raf kinase compared to wild type [19]. Taken together, these observations indicate that although the *RRAS2* duplication described in this study is an unconventional mutation for *ras* proto-oncogene activation, it is predicted to result in up-regulated MAPK pathway activity.

A previous study found *KRAS* mutation and *HER2* amplification to be almost mutually exclusive [1]. Although the number of cases we studied was smaller, we did not see this exclusivity: 2/6 HER2+ borderline and 3/6 HER2+ carcinomas also carried *KRAS* mutations. One caveat to this observation is that HER2 status in this study was based on IHC and/or high-level amplification (SNP array analysis) rather than a combined score including chromogenic in situ hybridization.

### Candidate mucinous ovarian cancer genes

In addition to the seven somatic *TP53* mutations identified by exome sequencing, Sanger sequencing of the DNA binding domain (exons 4–9) in the validation cohort identified a further 15 mutations at an overall frequency of 22/82 (26.8 %) MOTs, of which 21 were missense mutations (Table 2, Fig. 2). All 22 mutations have been previously reported in a somatic context (IARC *TP53* mutation database release 17). There was a significant difference in *TP53* mutation frequency among the three tumor subtypes (*P* = 0.003, Chi-square test). While there was a similar frequency of *TP53* mutations in benign (2/22, 9.1 %) and borderline (4/29, 13.8 %) tumors, 16/31 (51.6 %) of carcinomas harbored a *TP53* mutation (*P* = 0.002 and *P* = 0.002 compared to benign and borderline tumors respectively, Fisher’s exact test), suggesting that aberrant p53 contributes to the invasive phenotype in a proportion of these ovarian cancers. Both low-grade and high-grade carcinomas harbored mutations, which trended towards increasing frequency with grade (45.5 %, 53.8 % and 66.7 % in Grades 1, 2 and 3 respectively), and with an overall frequency similar to that of gastrointestinal mucinous carcinomas (Additional file 2: Figure S3). While it is well accepted that *TP53* mutation is an obligatory event in the genesis of high-grade serous ovarian carcinoma, we show by direct sequencing that mutant p53 is also common in mucinous-type ovarian carcinomas, but is a late event in their molecular progression. Interestingly, this group does not share the widespread genomic instability that typifies high-grade serous carcinomas that is contributed to, at least in part, by mutant *TP53*, suggesting different p53 activity in these two contexts.

**Fig. 3:**
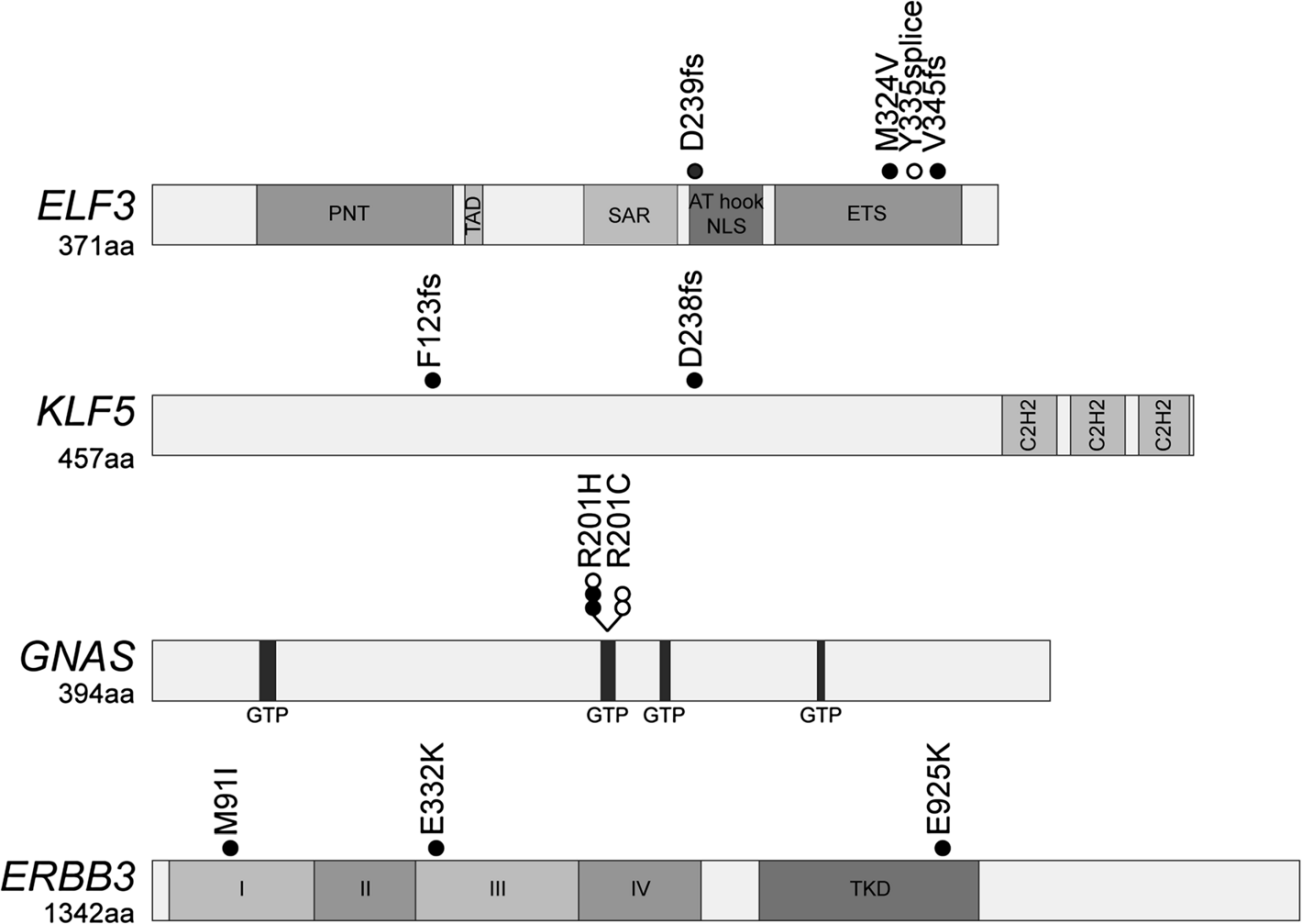
Distribution of somatic mutations identified in novel significantly mutated genes. *ELF3*, *KLF5*, *GNAS* and *ERBB3* are shown in the context of protein domains as predicted by UniProt, with somatic mutations identified in the exome (*closed circle*) and validation (*open circle*) cohorts mapped to each gene. *I-IV* extracellular domains I, II, III and IV, *AT hook & NLS* AT-hook domain and nuclear localization signal, *C2H2* zinc-finger C2H2 domain, *ETS* DNA binding domain, *GTP* GTP nucleotide binding region, *PNT* pointed domain, *SAR* serine-rich and aspartic acid-rich domain, *TAD* transactivation domain, *TKD* tyrosine kinase domain

Three mutations in the epithelial-specific ETS transcription factor *E74-like factor 3* (*ELF3*) were detected in three tumors by exome sequencing. *ELF3* was significantly mutated above background (MuSiC) and had an excess of likely deleterious mutations (OncodriveFM) including two frameshift insertions (p.Val345Glyfs*126, p.Asp239Glyfs*62) and a missense substitution (p.Met324Val) (Table 2, Figs. 2 and 3). Sequencing of the coding regions in the expanded cohort identified an additional splice site mutation in a borderline tumor (c.1001 + 1_1001 + 2insGG). Although *ELF3* is thus infrequently mutated (6.9 % borderline tumors and 6.5 % carcinomas), the shared characteristics of the four heterozygous mutations is indicative of a pathogenic role. Three of the mutations are overtly deleterious, including two frameshift indels and a canonical splice site mutation, while the missense mutation is predicted to be deleterious by computational analyses [20–22]. We further investigated the exon 8 splice donor site mutation by cDNA sequencing, which confirmed the use of an alternative donor splice sequence in the mutant allele (Additional file 2: Figure S4) consistent with the in silico prediction [23]. This mutation would result in out-of-frame, continued translation into the 3′-untranslated region (p.Tyr335Glyfs*113). cDNA sequencing of this and the two other truncating mutations found that all three mutations were readily detected in the tumor RNA, indicating that these mutations are not the subject of strong nonsense-mediated decay (Additional file 2: Figure S4). Truncating mutations in this epithelial-specific transcription factor have recently been reported in other cancers, including cancer of the cervix, stomach and bladder [24–26]. Interestingly, *ELF3*-mutated cervical carcinomas express ELF3 at a higher level compared to wild-type tumors [24]. This result may suggest that both copies of this gene are required and mutation of one allele results in up-regulation of the gene in an attempt to compensate. Alternatively, *ELF3* mutations may only have a selective advantage in tumors highly expressing ELF3.

*ELF3* has previously been identified as a candidate cancer gene; however, its role appears to be context dependent, in keeping with the tissue-specific nature of its transcriptional target genes. An oncogenic role has been suggested for breast cancer, with the gene being amplified and overexpressed [27, 28]. A positive feedback loop between ELF3 and HER2 exists in breast cancer, where ELF3 is both a downstream mediator and activator of HER2 signaling [29]. Of note in this study, two of two mutated carcinomas were HER2+, while the two mutated borderline tumors were HER2-. However, in a gastrointestinal tissue context, ELF3 may act as a tumor suppressor, because it is involved in positively transcriptionally regulating *TGFBR2*, facilitating the growth inhibitory consequences of TGF-β signaling [30, 31]. *ELF3* was identified as a cancer gene in a recent pan-cancer study, with enrichment for mutations in bladder and colorectal cancer [32]. The frequency of mutations in MOTs suggests that *ELF3* is indeed a cancer gene in this tumor type, but its exact role is unclear from the mutational profile—while the mutations are detrimental in nature, the retention of the wild-type allele argues against a classical tumor suppressor gene functional mechanism.

Another transcription factor with a proclivity for truncating mutations was *KLF5*, which encodes a zinc finger transcriptional activator (Table 2, Figs. 2 and 3). Exome sequencing identified two heterozygous frameshift mutations (p.Phe123Leufs*3 and p.Asp238Argfs*16); however, sequencing the coding region in a validation cohort of carcinomas failed to identify additional changes. Collectively, *KLF5* is mutated in 6.7 % (2/30) of mucinous ovarian carcinomas. Like *ELF3*, it has been identified as a pan-cancer gene but enriched for mutations in bladder, colorectal, and head and neck squamous carcinoma [32], and has been variously described as both an oncogene [33] and a tumor suppressor gene [34].

Considering exome and validation cohorts, five constitutively activating mutations at arginine codon 201 of the oncogene *GNAS* were identified, including 2/22 (9.1 %) benign cystadenomas, 2/29 (6.9 %) borderline tumors and 1/30 (3.3 %) carcinomas (Table 2, Figs. 2 and 3). Hotspot mutations in this guanine nucleotide-binding protein alpha subunit have recently been identified in other pre-malignant or non-aggressive mucinous-type tumors of gastrointestinal origin, albeit at a higher frequency (Additional file 2: Figure S3), including intraductal papillary mucinous neoplasm of the pancreas and bile duct [35, 36]; appendiceal mucinous neoplasms (and its associated pseudomyxoma peritonei) [37, 38]; and adenoma of the colon/rectum, stomach and small intestine [39, 40]. In this context, constitutive activation of *GNAS* through codon 201 mutation has been shown to increase levels of cAMP, resulting in prominent mucin production but not cell growth [38]. Consistent with previous reports, simultaneous *KRAS* mutations were present in four MOTs, although this association was not statistically significant. Thus, unlike gastrointestinal mucinous-type tumors, *GNAS* activation occurs only rarely in those involving the ovary.

Although human epidermal growth factor receptors have been implicated in MOT progression through amplification and overexpression of ERBB2 (HER2), activating mutations in other family members have not been previously described. Exome sequencing identified three *ERBB3* (HER3) mutations (a borderline tumor with concurrent mutations, and a carcinoma) (Table 2, Figs. 2 and 3), including two in the extracellular domain (p.Met91Ile and p.Glu332Lys) and one in the kinase domain (p.Glu925Lys). No additional mutations were identified in a validation screen of 19 carcinomas, giving a final frequency of 4.7 % in MOTs. Frequent *ERBB3* mutations have recently been reported in other cancer types, including those of the colon, gallbladder and stomach [41, 42]. Although *ERBB3* contains an impaired kinase domain, it is capable of ligand binding and preferentially hetrodimerizes with *ERBB2* to potently activate cellular signaling pathways [43]. Thus the *ERBB3* mutations described here are predicted to cooperate with *ERBB2* to promote ligand-independent oncogenic transformation, as functionally demonstrated for other kinase and extracellular mutations in this gene [41]. Of note, the *ERBB3* mutant carcinoma was also HER2+. We also identified a single somatic extracellular domain mutation in another ErbB receptor, *ERBB4* (p.Glu57Asp).

### Additional mutated candidate genes

We also identified somatic mutations in epigenetic regulatory genes, including the chromatin-remodeling factors *ARID1A* (1/5 benign MOTs and 1/11 carcinomas; a predicted significantly mutated gene) and *ARID2* (1/11 carcinomas) (Table 2, Fig. 2). Both genes are recognized suppressors of tumorigenesis in multiple cancer types [44, 45]. Consistent with this, the two *ARID1A* mutations result in protein truncation (p.Gln1894Profs*7 and p.Arg2116Thrfs*33). A further missense mutation was found in the Polycomb-group protein member *ASXL1*.

In addition to *ELF3* and *KLF5*, other genes implicated in the control of gene expression were collectively mutated in multiple samples. Three transcriptional co-regulatory proteins contained somatic mutations, including the consensus driver gene *BCL-6 corepressor* (*BCOR*; 2/11 carcinomas including splice donor and missense mutations) [45], and proposed pan-cancer drivers *NCOR2* (inframe indel in a benign tumor) and *ARHGAP35* (1/11 carcinomas) [46, 47]. Other genes involved in transcription also featured, such as a single mutation in *TAF1* that forms the large subunit of the transcription factor II D complex and facilitates the initiation of transcription by RNA polymerase II, and a missense mutation at the serine 34 hotspot of pre-mRNA splicing factor *U2AF1*, which has been shown to alter the cancer transcriptome [48]. The *GATA3* transcription factor was also mutated in one carcinoma.

One other important group of genes mutated in MOTs included those associated with ubiquitin-mediated protein degradation. As well as frequent deleterious mutations in the E3 ubiquitin ligase *RNF43* [8], the consensus cancer gene and tumor suppressor *FBXW7* is noteworthy [45], encoding for the substrate recognition component of SCF (complex of SKP1, CUL1 and F-box protein)-type ubiquitin ligases. Recurrent heterozygous mutations (1/8 borderline tumors and 1/11 carcinomas) (Table 2, Fig. 2) are predicted to result in proteins with impaired (p.Asp560Asn) or absent (p.Arg278*) substrate binding capability that dominantly interfere with wildtype protein through the intact dimerization domain. Interestingly, the mutant borderline tumor also harbored bi-allelic mutations in another SCF complex gene *CUL1*. We also identified a missense mutation in the E3 ubiquitin-protein ligase and cancer gene *UBR5* [46].

Other genes identified based on significance prediction and mutated in 2/24 MOTs by exome sequencing included leucine-rich repeat kinase 2 (*LRRK2*), the ribosomal gene transcription termination factor *TTF1*, and *LPHN3*, which encodes a member of the latrophilin subfamily of G protein-coupled receptors (Table 2, Fig. 2). *DCLK1*, a newly identified marker of transformed stem cells in the gut [49], was also mutated in 8.3 % of cases (Table 2, Fig. 2), and is a recurrent target for mutation in neoplasms of the stomach [25], appendix [37] and skin [50]. Clonal heterogeneity may be a feature of *DCLK1*, as we and others [25, 50] observed mutations at low allelic fractions. Validation in a larger cohort of samples is needed to interpret the role of these genes in mucinous ovarian tumorigenesis.

## Conclusions

Little is known about the genomics of mucinous ovarian carcinoma beyond the known cancer driver genes. Here, through mutation analysis, we provide insight into the somatically altered genes of MOTs, identifying many candidates not previously implicated in this disease, including a higher than expected proportion of carcinomas with *TP53* and *BRAF* mutations, as well as the prevalent RAS pathway mutations and loss of p16. Therapies for this relatively rare entity have focused on general chemotherapeutics currently used in ovarian cancer. These therapies show limited success in treating advanced mucinous disease and novel targeted therapies would be beneficial, especially for high-grade carcinoma.

Using exome sequencing we could resolve driver mutations in four of the six MOTs without a *KRAS* or *BRAF* oncogenic mutation. Two tumors were likely driven by alternative mechanisms for constitutive RAS signaling (*RRAS2* mutation and HER2 amplification) with both also harboring cooperating events in *TP53* and *CDKN2A*. Two further tumors may be explained by a truncation in *ARID1A*, and *ELF3* mutation plus homozygous *CDKN2A* loss, leaving only a benign and a borderline MOT unexplained. Given this, and the fact that a significant proportion of mutations in candidate drivers were identified among the carcinoma cohort, it is plausible that these genes represent cooperative mechanisms contributing to tumor progression rather than novel initiating events; the diversity of biological processes and pathways they involve hints at a high level of molecular heterogeneity in this contribution. This study provides a basis for understanding the diverse pathways targeted by somatic mutation in mucinous tumors of the ovary, although further functional work is required to elucidate the role of novel, less commonly affected genes with conflicting roles in the literature, such as *ELF3* and *KLF5*.

It is clear from this and other studies that the genes underlying MOTs are markedly different from other ovarian cancer subtypes. Genetic changes in the RAS/RAF pathway and concurrent loss of cell cycle regulation through aberrant p16 define MOTs. Some of the mutated genes we have observed have been seen in other tumor types, including in genes more commonly associated with tumors of the gastrointestinal tract, pancreas and endometrium, such as *RNF43*, *ELF3*, *ARID1A* and *GNAS*. Comparing MOTs to mucinous-type tumors from other organ sites reveals some genetic similarities, but also some striking differences (Additional file 2: Figure S3). Like MOTs, colorectal mucinous carcinomas are the only group in which frequent *KRAS* and *BRAF* mutations are found; mutations in both genes are absent in breast and rare gastric mucinous carcinomas, and appendiceal mucinous tumors are *BRAF* wild type. Likewise, pancreatic carcinomas and their mucinous neoplastic precursors appear not to be driven by oncogenic *BRAF*, but instead are the only group apart from MOTs to harbor mutant *CDKN2A*. We also identified genes novel to cancer that may reflect rarely targeted genes unique to the mucinous ovarian milieu. The initiating cell type of mucinous tumors presenting on the ovary remains to be determined; the heterogeneity of the mutations observed here as well as the mutational spectrum suggests that the ovarian surface epithelium is unlikely to be the only source.

## Additional files

Additional file 1: Table S1. Clinicopathological data for MOTs. **Table S2.** Summary of whole exome sequencing statistics. **Table S3.** List of all somatic coding mutations identified by whole exome sequencing and targeted Sanger sequencing. **Table S4.** Primers used for the validation screens of *ELF3*, *GNAS*, *KLF5* and *ERBB3*. **Table S5.** Mean mutation rates. **Table S6.** Somatic mutations identified in the validation cohort. (XLSX 208 kb) Additional file 2: Figure S1. Nucleotide substitution frequency and context. **Figure S2.** *RRAS2* somatic mutation. **Figure S3.** Genetic comparison between mucinous ovarian tumors and mucinous cancers from other anatomical sites. **Figure S4.** *ELF3* somatic mutations. **Figure S5.** H&E stained sections of frozen tissues used for exome discovery cohort. (PDF 9819 kb)
