# Erratum to: Mutational landscape of mucinous ovarian carcinoma and its neoplastic precursors

**DOI:** 10.1186/s13073-016-0392-y

**Published:** 2017-01-12

**Authors:** Georgina L. Ryland, Sally M. Hunter, Maria A. Doyle, Franco Caramia, Jason Li, Simone M. Rowley, Michael Christie, Prue E. Allan, Andrew N. Stephens, David D. L. Bowtell, Australian Ovarian Cancer Study Group, Ian G. Campbell, Kylie L. Gorringe

**Affiliations:** 1 Cancer Genetics Laboratory, Peter MacCallum Cancer Centre, East Melbourne, VIC Australia; 2 Bioinformatics Core Facility, Peter MacCallum Cancer Centre, East Melbourne, VIC Australia; 3 Department of Anatomical Pathology, Royal Melbourne Hospital, Parkville, VIC Australia; 4 Department of Pathology, Peter MacCallum Cancer Centre, East Melbourne, VIC Australia; 5 Centre for Cancer Research, MIMR-PHI Institute of Medical Research, Clayton, VIC Australia; 6 Department of Molecular and Translational Sciences, Monash University, Clayton, VIC Australia; 7 Epworth Research Institute, Epworth HealthCare, Richmond, VIC Australia; 8 Cancer Genetics and Genomics Laboratory, Peter MacCallum Cancer Centre, East Melbourne, VIC Australia; 9 Sir Peter MacCallum Department of Oncology, University of Melbourne, Parkville, VIC Australia; 10 Department of Biochemistry and Molecular Biology, University of Melbourne, Parkville, VIC Australia; 11 Department of Pathology, University of Melbourne, Parkville, VIC Australia

## Erratum

It has come to our attention that there is an error in Additional file 2 for this article [1]. When opening Additional file 2, this links to the incorrect file. The correct version of Additional file 2 can be found below. The publishers apologise for any inconvenience caused.

## Additional file

Additional file 2: Figure S1. Nucleotide substitution frequency and context. **Figure S2.** *RRAS2* somatic mutation. **Figure S3.** Genetic comparison between mucinous ovarian tumors and mucinous cancers from other anatomical sites. **Figure S4.** *ELF3* somatic mutations. **Figure S5.** H&E stained sections of frozen tissues used for exome discovery cohort. (PDF 9813 kb)
