# Supplementary material for: Mutational landscape of mucinous ovarian carcinoma and its neoplastic precursors
Source: Genome Med. 2015 Aug 7;7(1):87. doi: 10.1186/s13073-015-0210-y (PMC4528310; doi:10.1186/s13073-015-0210-y)
Supplement: Additional file 2: Figure S1. — Nucleotide substitution frequency and context. Figure S2. RRAS2 somatic mutation. Figure S3. Genetic comparison between mucinous ovarian tumors and mucinous cancers from other anatomical sites. Figure S4. ELF3 somatic mutations. Figure S5. H&E stained sections of frozen tissues used for exome discovery cohort. (PDF 9819 kb) [file 13073_2015_210_MOESM2_ESM.pdf]

Genome Medicine

Mutational landscape of mucinous ovarian carcinoma and its neoplastic precursors

--Manuscript Draft--

**Manuscript Number:**	GMED-D-15-00145R2
**Full Title:**	Mutational landscape of mucinous ovarian carcinoma and its neoplastic precursors
**Article Type:**	Research
**Funding Information:**	National Health and Medical Research Council (APP1045783) — Dr Kylie Louise Gorringe<br>National Health and Medical Research Council (628630) — Prof Ian G Campbell<br>Emer Casey Foundation (NA) — Prof Ian G Campbell<br>Victorian Breast Cancer Research Consortium (NA) — Prof Ian G Campbell<br>Medical Research and Materiel Command, U.S. Army Medical Department (DAMD17-01-1-0729) — Prof David DL Bowtell<br>The Cancer Council Tasmania (NA) — Prof David DL Bowtell<br>The Cancer Foundation of Western Australia (NA) — Prof David DL Bowtell<br>National Health and Medical Research Council (400413) — Prof David DL Bowtell<br>Ovarian Cancer Research Foundation (NA) — Dr Andrew N Stephens
**Abstract:**	Background: Mucinous ovarian tumors are an unusual group of rare neoplasms with an apparently clear progression from benign to borderline to carcinoma, yet with a controversial cell of origin in the ovarian surface epithelium. They are thought to be molecularly distinct from other ovarian tumors but there have been no exome-level sequencing studies performed to date.<br>Methods: To understand the genetic etiology of mucinous ovarian tumors and assess the presence of novel therapeutic targets or pathways, we undertook exome sequencing of 24 tumors encompassing benign (5), borderline (8) and carcinoma (11) histologies and also assessed a validation cohort of 58 tumors for specific gene regions including exons 4-9 of TP53.<br>Results: The predominant mutational signature was of C>T transitions in a NpCpG context indicative of deamination of methyl-cytosines. As well as mutations in known drivers (KRAS, BRAF and CDKN2A), we identified a high percentage of carcinomas with TP53 mutations (52%), and recurrent mutations in RNF43, ELF3, GNAS, ERBB3 and KLF5.<br>Conclusions: The diversity of mutational targets suggests multiple routes to tumorigenesis in this heterogeneous group of tumors that is generally distinct from other ovarian subtypes.
**Corresponding Author:**	Kylie L Gorringe, PhD<br>Peter MacCallum Cancer Centre<br>East Melbourne, VIC AUSTRALIA
**Corresponding Author Secondary Information:**	
**Corresponding Author's Institution:**	Peter MacCallum Cancer Centre
**Corresponding Author's Secondary Institution:**	
**First Author:**	Georgina L Ryland, PhD

**First Author Secondary Information:**	
**Order of Authors:**	Georgina L Ryland, PhD
	Sally M Hunter, PhD
	Maria A Doyle, PhD
	Franco Caramia
	Jason Li
	Simone M Rowley
	Michael Christie
	Prue E Allan
	Andrew N Stephens
	David DL Bowtell
	Ian G Campbell
	Kylie Louise Gorringe, PhD
**Order of Authors Secondary Information:**	
**Response to Reviewers:**	-Please add a Methods section to your abstract. For more information, please see: http://www.genomemedicine.com/authors/instructions/research#formatting-abstract<br>Done<br><br>-Please re-order you manuscript as: Background, Methods, Results, Discussion (or Results and Discussion), Conclusions. For more information, please see: http://www.genomemedicine.com/authors/instructions/research<br>Done<br><br>-Please add a statement regarding whether your study conforms to the Declaration of Helsinki.<br>Done (see methods)<br><br>-Please ensure that all data are publicly available and the accession numbers included in the Methods section. Data deposition must comply with your IRB approval, patient consent and confidentiality agreements.<br>Data available in Additional File 1 (methods adjusted to state this)

Manuscript
Click here to download Manuscript: Gorring ms for accept Tables corrected.docx
Click here to view linked References

Mutational landscape of mucinous ovarian carcinoma and its neoplastic precursors

Georgina L. Ryland[1], Sally M. Hunter[1], Maria A. Doyle[2], Franco Caramia[2], Jason Li[2], Simone M. Rowley[1], Michael Christie[3], Prue E. Allan[4], Andrew N. Stephens[5,6,7], David D. Bowtell[8,9,10], Australian Ovarian Cancer Study Group[11], Ian G. Campbell[1,9,12] and Kylie L. Gorringe[1,9,12]

1. Cancer Genetics Laboratory, Peter MacCallum Cancer Centre, East Melbourne, Victoria, Australia.

2. Bioinformatics Core Facility, Peter MacCallum Cancer Centre, East Melbourne, Victoria, Australia.

3. Department of Anatomical Pathology, Royal Melbourne Hospital, Parkville, Victoria, Australia.

4. Department of Pathology, Peter MacCallum Cancer Centre, East Melbourne, Victoria, Australia.

5. Centre for Cancer Research, MIMR-PHI Institute of Medical Research, Clayton, Victoria, Australia.

6. Department of Molecular and Translational Sciences, Monash University, Clayton, Victoria, Australia.

7. Epworth Research Institute, Epworth HealthCare, Richmond, Victoria, Australia.

8. Cancer Genetics and Genomics Laboratory, Peter MacCallum Cancer Centre, East Melbourne, Victoria, Australia.

9. Sir Peter MacCallum Department of Oncology, University of Melbourne, Parkville, Victoria, Australia.

10. Department of Biochemistry and Molecular Biology, University of Melbourne, Parkville, Victoria, Australia.

11. Australian Ovarian Cancer Study Group members and affiliations can be found at www.aocstudy.org.

12. Department of Pathology, University of Melbourne, Parkville, Victoria, Australia.

Running title: Mutational landscape of ovarian mucinous tumors

Grant Support: This study was supported by the Victorian Breast Cancer Research Consortium (VBCRC), the National Health and Medical Research Council of Australia (ID 628630) and The Emer Casey Foundation. The Australian Ovarian Cancer Study is supported by the U.S. Army Medical Research and Materiel Command under DAMD17-01-1-0729, The Cancer Council Tasmania and The Cancer Foundation of Western Australia and the National Health and Medical Research Council of Australia (ID 400413). A.N. Stephens is supported by a grant from the Ovarian Cancer Research Foundation.

Corresponding author: Kylie Gorringe, Cancer Genetics Laboratory, Peter MacCallum Cancer Centre, Locked Bag 1, A'Beckett St, Melbourne, VIC 8006, Australia. Phone +613 96561131; Fax +613 96561411; E-mail: kylie.gorringe@petermac.org.

Word count: 4869

Number of figures and tables: 4

Abstract

Background: Mucinous ovarian tumors are an unusual group of rare neoplasms with an apparently clear progression from benign to borderline to carcinoma, yet with a controversial cell of origin in the ovarian surface epithelium. They are thought to be molecularly distinct from other ovarian tumors but there have been no exome-level sequencing studies performed to date.

Methods: To understand the genetic etiology of mucinous ovarian tumors and assess the presence of novel therapeutic targets or pathways, we undertook exome sequencing of 24 tumors encompassing benign (5), borderline (8) and carcinoma (11) histologies and also assessed a validation cohort of 58 tumors for specific gene regions including exons 4-9 of *TP53*.

Results: The predominant mutational signature was of C>T transitions in a NpCpG context indicative of deamination of methyl-cytosines. As well as mutations in known drivers (*KRAS*, *BRAF* and *CDKN2A*), we identified a high percentage of carcinomas with *TP53* mutations (52%), and recurrent mutations in *RNF43*, *ELF3*, *GNAS*, *ERBB3* and *KLF5*.

Conclusions: The diversity of mutational targets suggests multiple routes to tumorigenesis in this heterogeneous group of tumors that is generally distinct from other ovarian subtypes.

Keywords: ovarian cancer, mucinous, exome sequencing, somatic, mutation

Background

Epithelial ovarian tumors have historically been treated as a homogenous group in the clinic, despite clear histo-pathological and molecular data showing that distinct subgroups exist: serous, endometrioid, clear cell and mucinous. High-grade serous and low-grade serous comprise distinct groups, while endometrioid and clear-cell histologies are different again from serous but with some overlapping genetic events. It is now clear that these molecular distinctions reflect differences in site of origin, with high-grade serous now thought to arise from the fallopian tube fimbriae, low-grade serous from the ovarian epithelium, and clear cell and endometrioid arising from endometriosis which itself is derived from the endometrium. However, the origin of the mucinous group remains controversial. Many ovarian mucinous tumors formerly classified as primary are now recognized to have been mis-diagnosed metastases from predominantly gastrointestinal or endocervical sites. However, some mucinous tumors do appear to be ovarian primaries, particularly benign and borderline tumors, which generally have a good prognosis not consistent with a metastatic tumor. Carcinomas associated with benign and borderline elements and/or with an early-stage, unilateral presentation are also thought to be primary ovarian in origin.

Our understanding of the genomic landscape of mucinous ovarian tumors (MOTs) is limited. Older reports are likely to include a high proportion of metastatic mucinous tumors, and the rarity of true primary mucinous tumors has limited investigations. Nonetheless, we and others have shown that mucinous tumors have a high proportion of mutations in RAS pathway genes and aberration of *CDKN2A* (p16) [1, 2]. Beyond these common drivers, little is known, and the predominantly stable genomic copy number profiles we have observed in this tumor type [3] suggest that somatic point mutations are likely to be more relevant. In this study, we have

undertaken exome sequencing of a large cohort of ovarian mucinous tumors, and have further investigated lead candidates in a validation cohort of 58 cases.

Methods

Specimens

Fresh-frozen MOTs were accessed from bio-banked specimens collected and cryopreserved at the time of surgical resection for a primary ovarian tumor, prior to chemotherapy administration, including 22 benign cystadenomas, 29 tumors of low malignant potential (herein referred to as borderline tumors) and 31 carcinomas [44, 45]. Hospitals contributing samples between 1993 and 2011 included those in the south of England, UK [45] and in Australia (Southern Health and the Australian Ovarian Cancer Study, AOCS [44]). Blood samples used for germline DNA extraction were also collected prior to surgery. Thorough histological classification was made based on the entire specimen at time of diagnosis although all cases underwent retrospective pathological review using information obtained from the pathology report and histological assessment according to established criteria [46] in order to exclude likely metastases. Cases were also excluded if there was insufficient tumour epithelium for nucleic acid extraction. Carcinoma grade was derived from the diagnostic pathology report as there were insufficient cases with archival specimens available for re-review. Clinicopathological data is provided in Additional File 1, Table S1.

This study was performed in accordance with the ethical standards of the Peter MacCallum Cancer Centre Human Research Ethics Committee (Approvals 09/29 and 01/38) and all participants provided written informed consent for tissue collection. This study conforms to the Declaration of Helsinki.

DNA extraction

Tumor genomic DNA was isolated by needle microdissection of areas with greater than 80% neoplastic cellularity from consecutive 10 µm H&E stained tumor sections and extracted using the DNeasy Blood and Tissue Kit (Qiagen) as per the recommended protocol. Matched germline DNA was extracted from whole blood (19 cases) or paired uninvolved ovarian stroma (5 cases). 20-50 ng of tumor and germline DNA underwent whole genome amplification (WGA) using the Repli-G Phi-mediated amplification system (Qiagen) and was used to confirm mutations detected by exome sequencing and to perform candidate gene mutation analysis.

Whole-exome library construction and sequencing

Libraries were constructed from 500 ng of unamplified tumor or germline DNA following the Illumina TruSeq DNA Sample Preparation procedure (Illumina), followed by exome capture using the NimbleGen SeqCap EZ Human Exome Library v1 or v2 capture kit (Roche NimbleGen). Each resulting paired-end library was sequenced on one-third of an Illumina HiSeq2000 lane using 75 bp or 100 bp reads. Library preparation and detailed summary statistics for all samples are listed in Additional File 1, Table S2.

Somatic mutation analysis

Purity filtered paired-end reads were quality checked with FastQC (v0.10.1) and trimmed for low quality bases and adaptor if necessary using Cutadapt (v1.1). Reads were then aligned to the human genome (GRCh37/hg19) using BWA-MEM (v0.7.7-r441). Duplicates were marked using Picard (v1.77) followed by local indel re-alignment and base quality score recalibration using GATK (v2.7-2-g6bda569). Somatic single nucleotide variants (SNVs) and insertions-deletions (indels) were called using the following algorithms with the matched

germline data used as reference: MuTect (v2.7-1-g42d771f), JointSNVMix (v0.8-b2) and Somatic Sniper (1.0.2.2-1-g8ee3999) (SNVs only), SomaticIndelDetector (v1.0.4905) (indels only), and VarScan (v2.3.4) (both SNVs and indels). In addition, Pindel (v0.2.5a3) and GATK Unified Genotyper (v2.7-2-g6bda569) were used to call SNVs and indels separately in the tumor and germline samples.

Initial variant predictions were filtered to require that i) SNVs were called by ≥2 of MuTect, JointSNVMix, Somatic Sniper, VarScan or Unified Genotyper, ii) indels were called by any of SomaticIndelDetector, VarScan, Pindel or Unified Genotyper, iii) the variant was present in ≥10 reads in the tumor (Pindel) or ≥2 reads in the tumor (all other callers), iv) the mutant allele frequency was ≤5% in the matched germline sample, and v) the mutant allele fraction was at least 10% higher in the tumor than in the matched germline sample for indels called by Pindel and Unified Genotyper. Finally, any remaining germline SNPs or common sequence artefacts were eliminated by requiring that the variant allele was not observed in more than 2 of the other germline samples from this cohort or more than 2 (of 147) in-house germline exome sequences [47], and had an Exome Variant Server (ESP6500 SI-v2) minor allele frequency of ≤5%.

Predicted somatic mutations were annotated with Ensembl v73 information and those with impact predictions overlapping coding regions and splice sites (±2 bp) were considered for further analysis. Due to restrictions of our ethics approval, we are not able to provide BAM files; however, all variants are available in Additional File 1, Table S3. All coding mutations were manually reviewed by examination of BAM files using the Integrative Genomics Viewer.

Mutation confirmation by nucleotide sequencing

Selected somatic mutations were independently assessed by PCR and Sanger sequencing of the tumor DNA as described previously [9]. Somatic status was confirmed by also resequencing the corresponding germline sample.

Thirty-two known somatic mutations in *KRAS*, *BRAF*, *TP53* and *CDKN2A* that had been independently validated in other studies of this cohort by Sanger sequencing were used to assess the sensitivity of somatic mutation calling, with 96.9% known somatic variants successfully identified (27/27 SNVs and 4/5 indels). Failure to identify a known 36 bp complex indel in *CDKN2A* was complicated by low read depth owing to high GC content for this gene; this variant was included in subsequent analyses. The confirmation rate of novel variants by Sanger sequencing was 93.6% (208/223 SNVs and 25/26 indels).

Significantly mutated gene prediction

The MuSiC algorithm (v0.4) was applied using default parameters to identify genes significantly enriched for mutations, given sequence type, context and estimated background rate [7]. Genes mutated in two or more samples with a p-value ≤0.05 at a false discovery rate of ≤0.1 in any of the three tests were considered significant. Furthermore, OncodriveFM (accessed through the online IntOGen platform at www.intogen.org) was used to identify genes with significant bias towards the accumulation of functional mutations (p-value ≤0.05 and q-value ≤0.1) [8, 48].

Mutation analysis of ELF3, ERBB3, GNAS, TP53, RAS-RAF and CDKN2A by Sanger sequencing

The complete coding exons of *ELF3* (exons 2-9) and *ERBB3* (exons 1-28) were assessed by direct Sanger sequencing using the primers listed in Additional File 1 Table S4. To assess the *ELF3* c.1001+1_1001+2insGG mutation on mRNA splicing, cDNA amplification and direct sequencing were performed using primers listed in Table S4. Targeted Sanger sequencing of mutation hotspots in *TP53* (exons 4-9), *BRAF* (codon 600), *KRAS*/*HRAS*/*NRAS* (codons 12, 13 and 61), and the coding region of *CDKN2A* (exons 1-3) were sequenced using primers previously described [2]. Somatic mutations identified in these genes have previously been published for a subset of the benign and borderline mucinous tumors [2].

Mutation analysis of GNAS and KLF5 by high-resolution melt analysis

High resolution melt analysis was used to screen for mutations at hotspot codon 201 of *GNAS* and in the coding exons of *KLF5* (exons 1-4). 15 ng WGA tumor DNA was amplified in duplicate using the primers listed in Additional File 1, Table S4, followed by melt analysis on the LightCycler 480 Instrument using Gene Scanning Software (Roche). Samples with variant melt curves in duplicate PCRs were independently amplified using the same primers (*KLF5*) or an independent primer set (*GNAS*) and Sanger sequenced to confirm sequence variations.

Analysis of CDKN2A and HER2

HER2 status was ascertained based on detection of high-level gene amplification by high-density genome-wide SNP arrays (Affymetrix) (35 cases) [2, 3], SNP array plus immunohistochemistry (IHC) (23 cases) or IHC alone (18 cases). For 16 cases, HER2 IHC was evaluated on 4 µM formalin fixed paraffin embedded whole sections using anti-HER2 antibody clone 4B5 (Ventana Medical Systems, USA). Staining was scored visually according to standard guidelines [49]; briefly, an IHC score of 3+ (strong uniform membrane staining of >30% tumor cells) was categorized as HER2 positive, equivocal cases (score of

2+, strong complete membrane staining in <30% tumor cells or weak to moderate heterogeneous staining in >10% tumor cells) were only considered HER2 positive if accompanied by array based copy number amplification of the *ERBB2* locus. An equivocal score without amplification confirmation, and tumors that scored 0 or 1 (no staining or weak incomplete membrane staining in any proportion of tumor cells), were considered negative. For the remaining 25 cases, HER2 IHC score was derived from Anglesio *et al*. [1], who used comparable classification guidelines.

Results and Discussion

Somatic mutation frequency and spectra

To profile the somatic mutation spectrum of mucinous tumors of the ovary, we performed whole exome sequencing on 24 tumors including 5 benign cystadenomas, 8 borderline tumors and 11 carcinomas (Table 1, Additional File 1, Table S1). A mean coverage depth of 144x was achieved in both neoplastic and non-cancerous specimens (range 53-fold to 224-fold) and of the exons, 91% were covered by at least 20 uniquely mapping reads (Additional File 1, Table S2). Using stringent criteria, 1126 somatic coding and essential splice site mutations were identified (1031 SNVs and 95 indels), of which 841 were predicted to alter protein sequence (Additional File 1, Table S3). These included 44 (5.2%) nonsense, 60 (7.1%) frameshift indel, 16 (1.9%) splice site mutations, 27 (3.2%) inframe indel and 694 (82.5%) missense. Benign and borderline tumors had on average 25.4 (range 21-38) and 32.9 (range 2-76) coding mutations per tumor equating to a frequency of 0.8 mutations/Mb and 0.9 mutations/Mb respectively. Although variable, this mutation burden did not differ between benign and borderline tumors but was significantly lower when compared to the carcinomas (average of 66.9 mutations per sample and 1.5 mutations/Mb) (p=0.008 and p=0.047 respectively), attributed mostly to an accumulation of missense mutations in the latter (Fig.

1a, Additional File 1, Table S5). There were no hyper-mutated cases (defined as >10 mutations/Mb) indicative of a mutator phenotype such as mismatch repair deficiency. Relative to other cancer types, MOTs showed a similar somatic mutation density to breast, serous ovarian and pancreatic cancers, but lower than colorectal and stomach tumors [4, 5].

The mutation spectrum was dominated by C>T transitions, comprising 63.9% of somatic substitutions, and this was common to all three tumor subtypes (Fig. 1b). Mutations in this context demonstrated a marked preference for NpCpG trinucleotides (Additional File 2, Fig. S1a), the optimum motif for spontaneous 5-methylcytosine deamination [6]. An equivalent signature is frequently seen in other epithelial tumors of the gastrointestinal tract, but is different to that observed in other cancers of the female reproductive system including high-grade serous ovarian carcinoma (Additional File 2, Fig. S1b). Taken together these findings are consistent with ovarian mucinous tumors having a shared lineage distinct from that of other ovarian epithelial tumors.

Profile of mutated genes in ovarian mucinous tumors

Protein altering mutations were detected in 761 genes, of which 42 were mutated in two or more of the 24 tumors. Among the most frequently mutated were known mucinous ovarian cancer genes *KRAS*, *BRAF* and *CDKN2A* (Table 2, Fig. 2). Interestingly, *TP53* was the second most frequently mutated gene, with seven mutations identified. Eight genes were significantly mutated based on a statistically significant accumulation of mutations by both MuSiC [7] and OncodriveFM [8] (Table 2). Other genes predicted by one algorithm are also notable; for example *ERBB3* (MuSiC) and *GNAS* and *FBXW7* (OncodriveFM) (Table 2). Based on these predictions and observation in other cancer types, five novel candidate drivers not previously reported in ovarian mucinous tumors were selected for validation in an

independent cohort – *TP53* (7/24), *ELF3* (3/24), *ERBB3* (2/24), *GNAS* (2/24) and *KLF5* (2/24) (Table 2, Fig. 2, Additional File 1, Table S6). Our validation study of the tumor suppressor gene *RNF43* has been published previously [9]. In addition, known cancer genes for this ovarian subtype were evaluated in parallel to assess their relationship with new drivers including HER2 (by immunohistochemistry and copy number analysis) and mutations in *KRAS*, *BRAF*, *CDKN2A* and other RAS pathway members *NRAS* and *HRAS*.

Prevalence of mutations in known mucinous ovarian cancer genes

We and others have previously described the importance of the RAS pathway and p16 in MOTs [2, 1, 3]. Here we extend this analysis and found 56 cases with mutations in *KRAS, BRAF* and *NRAS* (68.3%). *BRAF* mutations were significantly more prevalent in the carcinomas (7/31, 22.6%) than the borderline (3/29, 10.3%) or benign tumors (0/22, p=0.036 Fisher's exact test) suggesting an association with a more aggressive phenotype. An alternative mechanism for activation of the MAPK pathway was identified through mutation of the *ras*-like gene *RRAS2* in one benign tumor that was *KRAS*/*BRAF* wildtype (Fig. 2). We previously reported *RRAS2* gene amplification in this sample [2]; consistent with this, the Sanger sequencing validation confirmed homozygous amplification of the mutant allele (Additional File 2, Fig. S2). This 9 bp duplication, resulting in reiteration of Gly-Gly-Gly (codons 22-24), occurs in the region of *RRAS2* that is complementary to codons 11-13 within the G1 phosphate-binding loop of conventional ras proteins (P-loop, amino acids 10-17). Interestingly, rare reports of comparable events appear in the literature. Huang *et al*. [10] described a three amino acid *RRAS2* duplication (Gly24_26dup) in the human uterine leiomyosarcoma cell line ST-UT-1; this mutation resulted in enhanced GTP-binding and conferred transforming activity *in vitro*. Similarly, in *KRAS*, 9 bp and 12 bp tandem repeats of codons 10-12 and 10-13 respectively were identified as an alternative mechanism for *KRAS*

oncogenic activation in 2 of 18 chemically induced rat renal mesenchymal tumors [11]. Triple residue insertions in the P-loop of *HRAS* also demonstrate increased preference for GTP-binding and increased interactions with downstream Raf kinase compared to wildtype [12]. Taken together, these observations indicate that although the *RRAS2* duplication described in this study is an unconventional mutation for *ras* proto-oncogene activation, it is predicted to result in up-regulated MAPK pathway activity.

A previous study found *KRAS* mutation and *HER2* amplification to be almost mutually exclusive [1]. Although the number of cases studied here is smaller, we do not see this exclusivity: 2/6 HER2+ borderline and 3/6 HER2+ carcinomas also carried *KRAS* mutations. One caveat to this observation is that HER2 status in this study is based on immunohistochemistry and/or high-level amplification (SNP array analysis) rather than a combined score including CISH.

Candidate mucinous ovarian cancer genes

In addition to the seven somatic *TP53* mutations identified by exome sequencing, Sanger sequencing of the DNA binding domain (exons 4-9) in the validation cohort identified a further 15 mutations at an overall frequency of 22/82 (26.8%) MOTs, of which 21 were missense mutations (Table 2, Fig. 2). All 22 mutations have been previously reported in a somatic context (IARC *TP53* mutation database release 17). There was a significant difference in *TP53* mutation frequency among the three tumor subtypes (p=0.003, Chi-square test). While benign and borderline tumors were mutated at a similar frequency (2/22, 9.1% and 4/29, 13.8% respectively), 51.6% (16/31) of carcinomas harbored a *TP53* mutation (p=0.002 and p=0.002 compared to benign and borderline tumors respectively, Fisher's exact test), suggesting that aberrant p53 contributes to the invasive phenotype in a proportion of

these ovarian cancers. Both low- and high-grade carcinomas harbored mutations, which trended towards increasing frequency with grade (45.5%, 53.8% and 66.7% in Grades 1, 2 and 3 respectively), and with an overall frequency similar to that of gastrointestinal mucinous carcinomas (Additional File 2, Fig. S3). While it is well accepted that *TP53* mutation is an obligatory event in the genesis of high-grade serous ovarian carcinoma, we show by direct sequencing that mutant p53 is also common in mucinous-type ovarian carcinomas, but is a late event in their molecular progression. Interestingly, this group does not share the widespread genomic instability that typifies high-grade serous carcinomas which is contributed to, at least in part, by mutant *TP53*, suggesting different p53 activity in these two contexts.

Three mutations in the epithelial-specific ETS transcription factor *E74-like factor 3* (*ELF3*) were detected in three tumors by exome sequencing. *ELF3* was significantly mutated above background (MuSiC) and had an excess of likely deleterious mutations (OncodriveFM) including two frameshift insertions (p.Val345Glyfs*126, p.Asp239Glyfs*62) and a missense substitution (p.Met324Val) (Table 2, Fig. 2, Fig. 3). Sequencing of the coding regions in the expanded cohort identified an additional splice site mutation in a borderline tumor (c.1001+1_1001+2insGG). Although *ELF3* is thus infrequently mutated (6.9% borderline tumors and 6.5% carcinomas), the shared characteristics of the four heterozygous mutations is indicative of a pathogenic role. Three of the mutations are overtly deleterious, including two frameshift indels and a canonical splice site mutation, while the missense mutation is predicted to be deleterious by computational analyses [13-15]. We further investigated the exon 8 splice donor site mutation by cDNA sequencing, which confirmed the use of an alternative donor splice sequence in the mutant allele (Additional File 2, Fig. S4) consistent with *in silico* prediction [16]. This mutation would result in out-of-frame, continued

translation into the 3'-untranslated region (p.Tyr335Glyfs*113). cDNA sequencing of this and the two other truncating mutations found that all three mutations were readily detected in the tumour RNA, indicating that these mutations are not the subject of strong nonsense mediated decay (Additional File 2, Fig. S4). Truncating mutations in this epithelial-specific transcription factor have recently been reported in other cancers, including cancer of the cervix, stomach and bladder [17-19]. Interestingly, *ELF3*-mutated cervical carcinomas express ELF3 at a higher level compared to wildtype tumors [17]. This result may suggest that both copies of this gene are required and mutation of one allele results in upregulation of the gene in an attempt to compensate. Alternatively, *ELF3* mutations may only have a selective advantage in tumors highly expressing ELF3.

ELF3 has previously been identified as a candidate cancer gene, however its role appears to be context dependent, in keeping with the tissue-specific nature of its transcriptional target genes. An oncogenic role has been suggested for breast cancer, with the gene being amplified and overexpressed [20, 21]. A positive feedback loop between ELF3 and HER2 exists in breast cancer, where ELF3 is both a downstream mediator and activator of HER2 signaling [22]. Of note in this study, 2/2 mutated carcinomas were HER2+, while the two mutated borderline tumors were HER2-. However, in a gastrointestinal tissue context ELF3 may act as a tumor suppressor, as it is involved in positively transcriptionally regulating *TGFBR2*, facilitating the growth inhibitory consequences of TGF-β signaling [23, 24]. *ELF3* was identified as a cancer gene in a recent pan-cancer study, with enrichment for mutations in bladder and colorectal cancer [25]. The frequency of mutations in MOTs suggests that *ELF3* is indeed a cancer gene in this tumor type, but its exact role is unclear from the mutational profile – while the mutations are detrimental in nature, the retention of the wildtype allele argues against a classical tumor suppressor gene functional mechanism.

Another transcription factor with a proclivity for truncating mutations was *KLF5*, which encodes a zinc finger transcriptional activator (Table 2, Fig. 2 and Fig. 3). Exome sequencing identified two heterozygous frameshift mutations (p.Phe123Leufs*3 and p.Asp238Argfs*16), however, sequencing the coding region in a validation cohort of carcinomas failed to identify additional changes. Collectively, *KLF5* is mutated in 6.7% (2/30) of mucinous ovarian carcinomas. Like *ELF3*, it has been identified as a pan-cancer gene but enriched for mutations in bladder, colorectal and head and neck squamous carcinoma [25], and has been variously described as both an oncogene [26] and a tumor suppressor gene [27].

Considering exome and validation cohorts, five constitutively activating mutations at arginine codon 201 of the oncogene *GNAS* were identified, including 2/22 (9.1%) benign cystadenomas, 2/29 (6.9%) borderline tumors and 1/30 (3.3%) carcinomas (Table 2, Fig. 2, Fig. 3). Hotspot mutations in this guanine nucleotide-binding protein alpha subunit have recently been identified in other pre-malignant or non-aggressive mucinous-type tumors of gastrointestinal origin, albeit at a higher frequency (Additional File 2, Fig. S3), including intraductal papillary mucinous neoplasm of the pancreas and bile duct [28, 29], appendiceal mucinous neoplasms (and its associated pseudomyxoma peritonei) [30, 31] and adenoma of the colorectum, stomach and small intestine [32, 33]. In this context, constitutive activation of *GNAS* through codon 201 mutation has been shown to increase levels of cAMP resulting in prominent mucin production, but not cell growth [31]. Consistent with previous reports, simultaneous *KRAS* mutations were present in four MOTs, although this association was not statistically significant. Thus, unlike gastrointestinal mucinous-type tumors, *GNAS* activation occurs only rarely in those involving the ovary.

Although human epidermal growth factor receptors have been implicated in MOT progression through amplification and overexpression of ERBB2 (HER2), activating mutations in other family members have not been previously described. Exome sequencing identified three *ERBB3* (HER3) mutations (a borderline tumor with concurrent mutations, and a carcinoma) (Table 2, Fig.2 and Fig. 3), including two in the extracellular domain (p.Met91Ile and p.Glu332Lys) and one in the kinase domain (p.Glu925Lys). No additional mutations were identified in a validation screen of 19 carcinomas, giving a final frequency of 4.7% in MOTs. Frequent *ERBB3* mutations have recently been reported in other cancer types including those of the colon, gallbladder, and stomach [34, 35]. Although *ERBB3* contains an impaired kinase domain, it is capable of ligand binding and preferentially hetrodimerizes with *ERBB2* to potently activate cellular signaling pathways [36]. Thus the *ERBB3* mutations described here are predicted to cooperate with *ERBB2* to promote ligand-independent oncogenic transformation, as functionally demonstrated for other kinase and extracellular mutations in this gene [34]. Of note, the *ERBB3* mutant carcinoma was also HER2+. We also identified a single somatic extracellular domain mutation in another ErbB receptor, *ERBB4* (p.Glu57Asp).

Additional mutated candidate genes

We also identified somatic mutations in epigenetic regulatory genes, including the chromatin-remodeling factors *ARID1A* (1/5 benign MOTs and 1/11 carcinomas; a predicted significantly mutated gene) and *ARID2* (1/11 carcinomas) (Table 2, Fig. 2). Both genes are recognized suppressors of tumorigenesis in multiple cancer types [37, 38]. Consistent with this, the two *ARID1A* mutations result in protein truncation (p.Gln1894Profs*7 and p.Arg2116Thrfs*33). A further missense mutation was found in the Polycomb-group protein member *ASXL1*.

In addition to *ELF3* and *KLF5*, other genes implicated in the control of gene expression were collectively mutated in multiple samples. Three transcriptional co-regulatory proteins contained somatic mutations including the consensus driver gene *BCL-6 corepressor* (*BCOR*; 2/11 carcinomas including splice donor and missense mutations) [38] and proposed pan-cancer drivers *NCOR2* (inframe indel in a benign tumor) and *ARHGAP35* (1/11 carcinomas) [39, 40]. Other genes involved in transcription also featured, such as a single mutation in *TAF1* that forms the large subunit of the transcription factor II D complex and facilitates the initiation of transcription by RNA polymerase II, and a missense mutation at the serine 34 hotspot of pre-mRNA splicing factor *U2AF1*, which has been shown to alter the cancer transcriptome [41]. The *GATA3* transcription factor was also mutated in one carcinoma.

One other important group of genes mutated in MOTs included those associated with ubiquitin-mediated protein degradation. As well as frequent deleterious mutations in the E3 ubiquitin ligase *RNF43* [9], the consensus cancer gene and tumor suppressor *FBXW7* is noteworthy [38], encoding for the substrate recognition component of SCF (complex of SKP1, CUL1 and F-box protein)-type ubiquitin ligases. Recurrent heterozygous mutations (1/8 borderline tumors and 1/11 carcinomas) (Table 2, Fig. 2) are predicted to result in proteins with impaired (p.Asp560Asn) or absent (p.Arg278*) substrate binding capability that dominantly interfere with wildtype protein through the intact dimerization domain. Interestingly, the mutant borderline tumor also harbored bi-allelic mutations in another SCF complex gene *CUL1*. We also identified a missense mutation in the E3 ubiquitin-protein ligase and cancer gene *UBR5* [39].

Other genes identified based on significance prediction and mutated in 2/24 MOTs by exome sequencing include leucine-rich repeat kinase 2 (*LRRK2*), the ribosomal gene transcription

termination factor *TTF1*, and *LPHN3*, which encodes a member of the latrophilin subfamily of G protein-coupled receptors (Table 2, Fig. 2). *DCLK1*, a newly identified marker of transformed stem cells in the gut [42], was also mutated in 8.3% of cases (Table 2, Fig. 2), and is a recurrent target for mutation in neoplasms of the stomach [18], appendix [30] and skin [43]. Clonal heterogeneity may be a feature of *DCLK1*, as we and others [43, 18] observed mutations at low allelic fractions. Validation in a larger cohort of samples is needed to interpret the role of these genes in mucinous ovarian tumorigenesis.

Conclusions

Little is known about the genomics of mucinous ovarian carcinoma beyond the known cancer driver genes. Here, through mutation analysis, we provide insight into the somatically altered genes of MOTs, identifying many candidates not previously implicated in this disease, including a higher than expected proportion of carcinomas with *TP53* and *BRAF* mutations, as well as the prevalent RAS pathway mutations and loss of p16. Therapies for this relatively rare entity have focused on general chemotherapeutics currently used in ovarian cancer. These therapies show limited success in treating advanced mucinous disease and novel targeted therapies would be beneficial, especially for high-grade carcinoma.

Using exome sequencing we could resolve driver mutations in four of the six MOTs without a *KRAS* or *BRAF* oncogenic mutation. Two tumors are likely driven by alternative mechanisms for constitutive RAS signaling (*RRAS2* mutation and HER2 amplification) with both also harboring cooperating events in *TP53* and *CDKN2A*. Two further tumors may be explained by a truncation in *ARID1A*, and *ELF3* mutation plus homozygous *CDKN2A* loss, leaving only a benign and a borderline MOT unexplained. Given this, and the fact that a significant fraction of mutations in candidate drivers were identified among the carcinoma cohort, it’s plausible

that these genes represent cooperative mechanisms contributing to tumor progression rather than novel initiating events; the diversity of biological processes and pathways they involve hints at a high level of molecular heterogeneity in this contribution. This study provides a basis for understanding the diverse pathways targeted by somatic mutation in mucinous tumors of the ovary, although further functional work is required to elucidate the role of novel, less commonly effected genes with conflicting roles in the literature, such as *ELF3* and *KLF5*.

It is clear from this and other studies that the genes underlying MOTs are markedly different from other ovarian cancer subtypes. Genetic changes in the RAS/RAF pathway and concurrent loss of cell cycle regulation through aberrant p16 define MOTs. Some of the mutated genes we have observed have been seen in other tumor types, including in genes more commonly associated with tumors of the gastrointestinal tract, pancreas and endometrium, such as *RNF43*, *ELF3*, *ARID1A* and *GNAS*. Comparing MOTs to mucinous-type tumors from other organ sites reveals some genetic similarities, but also some striking differences (Additional File 2, Fig. S3). Like MOTs, colorectal mucinous carcinomas are the only group in which frequent *KRAS* and *BRAF* mutations are found; mutations in both genes are absent in breast and rare gastric mucinous carcinomas, and appendiceal mucinous tumors are *BRAF* wildtype. Likewise, pancreatic carcinomas and their mucinous neoplastic precursors appear not to be driven by oncogenic *BRAF*, but instead are the only group apart from MOTs to harbor mutant *CDKN2A*. We also identified genes novel to cancer that may reflect rarely targeted genes unique to the mucinous ovarian milieu. The initiating cell type of mucinous tumors presenting on the ovary remains to be determined; the heterogeneity of the mutations observed here as well as the mutational spectrum suggests that the ovarian surface epithelium is unlikely to be the only source.

Competing Interests: The authors declare no potential conflicts of interest.

Author Contributions:

GLR, IGC and KLG were involved in the conception and design of the study. AOCS, DDLB and ANS enrolled and managed patients; MC and PEA undertook pathology review; GLR, SMH and SMR performed the laboratory experiments. Statistical and bioinformatics analyses were undertaken by GLR, SMH, MAD, FC, JL and KLG. GLR, SMH, IGC and KLG drafted the manuscript. All authors read and revised the manuscript.

Additional data files:

The following additional data are available with the online version of this paper: Additional file 1.xlsx is a set of 6 Excel spreadsheets containing Table S1 (clinicopathological data), Table S2 (whole exome sequencing summary), Table S3 (somatic mutations identified in discovery cohort), Table S4 (primer sequences), Table S5 (mean mutation rates), Table S6 (somatic mutations identified in validation cohort). Additional file 2.doc contains Figure S1 (nucleotide substitution frequency and context), Figure S2 (RRAS2 mutation), Figure S3 (comparison of mutation frequencies between mucinous tumors from various anatomical sites), and Figure S4 (ELF3 mutations).

Acknowledgements

The authors gratefully acknowledge the cooperation of the institutions in Australia participating in the Australian Ovarian Cancer Study (AOCS). We also acknowledge the contribution of the AOCS study nurses, research assistants and all clinical and scientific collaborators and would like to thank all of the women who participated in AOCS. Members of the Australian Ovarian Cancer Study Group, collaborators and hospitals involved in AOCS

can be found at www.aocstudy.org. The authors also thank A/Prof Thomas W. Jobling (Department of Obstetrics and Gynaecology, Monash Medical Centre, Clayton) and the Ovarian Cancer Research Foundation (www.OCRF.com.au) for supporting the collection of patient samples and information used in this study.

References

1. Anglesio MS, Kommoss S, Tolcher MC, Clarke B, Galletta L, Porter H et al. Molecular characterization of mucinous ovarian tumours supports a stratified treatment approach with HER2 targeting in 19% of carcinomas. J Pathol. 2013;229(1):111-20. doi:10.1002/path.4088.
2. Hunter SM, Gorringe KL, Christie M, Rowley SM, Bowtell DD, Campbell IG. Pre-invasive ovarian mucinous tumors are characterized by *CDKN2A* and ras pathway aberrations. Clin Cancer Res. 2012;18(19):5267-77. doi:10.1158/1078-0432.CCR-12-1103.
3. Gorringe KL, Ramakrishna M, Williams LH, Sridhar A, Boyle SE, Bearfoot JL et al. Are there any more ovarian tumor suppressor genes? A new perspective using ultra high-resolution copy number and loss of heterozygosity analysis. Genes Chromosomes Cancer. 2009;48(10):931-42. doi:10.1002/gcc.20694.
4. Lawrence MS, Stojanov P, Mermel CH, Robinson JT, Garraway LA, Golub TR et al. Discovery and saturation analysis of cancer genes across 21 tumour types. Nature. 2014;505(7484):495-501. doi:10.1038/nature12912.
5. Lawrence MS, Stojanov P, Polak P, Kryukov GV, Cibulskis K, Sivachenko A et al. Mutational heterogeneity in cancer and the search for new cancer-associated genes. Nature. 2013;499(7457):214-8. doi:10.1038/nature12213.
6. Laird PW, Jaenisch R. DNA methylation and cancer. Hum Mol Genet. 1994;3(Suppl 1):1487-95.
7. Dees ND, Zhang Q, Kandoth C, Wendl MC, Schierding W, Koboldt DC et al. MuSiC: identifying mutational significance in cancer genomes. Genome Res. 2012;22(8):1589-98. doi:10.1101/gr.134635.111.
8. Gonzalez-Perez A, Lopez-Bigas N. Functional impact bias reveals cancer drivers. Nucleic Acids Res. 2012;40(21):e169. doi:10.1093/nar/gks743.
9. Ryland GL, Hunter SM, Doyle MA, Rowley SM, Christie M, Allan PE et al. *RNF43* is a tumour suppressor gene mutated in mucinous tumours of the ovary. J Pathol. 2013;229(3):469-76. doi:10.1002/path.4134.
10. Huang Y, Saez R, Chao L, Santos E, Aaronson SA, Chan AM. A novel insertional mutation in the TC21 gene activates its transforming activity in a human leiomyosarcoma cell line. Oncogene. 1995;11(7):1255-60.
11. Higinbotham KG, Rice JM, Buzard GS, Perantoni AO. Activation of the K-ras gene by insertion mutations in chemically induced rat renal mesenchymal tumors. Oncogene. 1994;9(9):2455-9.
12. Klockow B, Ahmadian MR, Block C, Wittinghofer A. Oncogenic insertional mutations in the P-loop of Ras are overactive in MAP kinase signaling. Oncogene. 2000;19(47):5367-76. doi:10.1038/sj.onc.1203909.
13. Adzhubei IA, Schmidt S, Peshkin L, Ramensky VE, Gerasimova A, Bork P et al. A method and server for predicting damaging missense mutations. Nat Methods. 2010;7(4):248-9. doi:10.1038/nmeth0410-248.
14. Ng PC, Henikoff S. Predicting deleterious amino acid substitutions. Genome Res. 2001;11(5):863-74. doi:10.1101/gr.176601.
15. Reva B, Antipin Y, Sander C. Predicting the functional impact of protein mutations: application to cancer genomics. Nucleic Acids Res. 2011. doi:10.1093/nar/gkr407.
16. Desmet FO, Hamroun D, Lalande M, Collod-Beroud G, Claustres M, Beroud C. Human Splicing Finder: an online bioinformatics tool to predict splicing signals. Nucleic Acids Res. 2009;37(9):e67. doi:gkp215 [pii]
10.1093/nar/gkp215.

17. Ojesina AI, Lichtenstein L, Freeman SS, Pedamallu CS, Imaz-Rosshandler I, Pugh TJ et al. Landscape of genomic alterations in cervical carcinomas. Nature. 2014;506(7488):371-5. doi:10.1038/nature12881.
18. Wang K, Yuen ST, Xu J, Lee SP, Yan HH, Shi ST et al. Whole-genome sequencing and comprehensive molecular profiling identify new driver mutations in gastric cancer. Nat Genet. 2014. doi:10.1038/ng.2983.
19. Nordentoft I, Lamy P, Birkenkamp-Demtroder K, Shumansky K, Vang S, Hornshoj H et al. Mutational context and diverse clonal development in early and late bladder cancer. Cell Rep. 2014;7(5):1649-63. doi:10.1016/j.celrep.2014.04.038.
20. Naylor TL, Greshock J, Wang Y, Colligon T, Yu QC, Clemmer V et al. High resolution genomic analysis of sporadic breast cancer using array-based comparative genomic hybridization. Breast Cancer Res. 2005;7(6):R1186-98.
21. Neve RM, Chin K, Fridlyand J, Yeh J, Baehner FL, Fevr T et al. A collection of breast cancer cell lines for the study of functionally distinct cancer subtypes. Cancer Cell. 2006;10(6):515-27. doi:10.1016/j.ccr.2006.10.008.
22. Neve RM, Ylstra B, Chang CH, Albertson DG, Benz CC. ErbB2 activation of ESX gene expression. Oncogene. 2002;21(24):3934-8. doi:10.1038/sj.onc.1205503.
23. Kopp JL, Wilder PJ, Desler M, Kim JH, Hou J, Nowling T et al. Unique and selective effects of five Ets family members, Elf3, Ets1, Ets2, PEA3, and PU.1, on the promoter of the type II transforming growth factor-beta receptor gene. J Biol Chem. 2004;279(19):19407-20. doi:10.1074/jbc.M314115200.
24. Flentjar N, Chu PY, Ng AY, Johnstone CN, Heath JK, Ernst M et al. TGF-betaRII rescues development of small intestinal epithelial cells in Elf3-deficient mice. Gastroenterology. 2007;132(4):1410-9. doi:S0016-5085(07)00413-1 [pii]
10.1053/j.gastro.2007.02.054.
25. Leiserson MD, Vandin F, Wu HT, Dobson JR, Eldridge JV, Thomas JL et al. Pan-cancer network analysis identifies combinations of rare somatic mutations across pathways and protein complexes. Nat Genet. 2015;47(2):106-14. doi:10.1038/ng.3168.
26. Nandan MO, McConnell BB, Ghaleb AM, Bialkowska AB, Sheng H, Shao J et al. Kruppel-like factor 5 mediates cellular transformation during oncogenic KRAS-induced intestinal tumorigenesis. Gastroenterology. 2008;134(1):120-30. doi:10.1053/j.gastro.2007.10.023.
27. Bateman NW, Tan D, Pestell RG, Black JD, Black AR. Intestinal tumor progression is associated with altered function of KLF5. J Biol Chem. 2004;279(13):12093-101. doi:10.1074/jbc.M311532200.
28. Sasaki M, Matsubara T, Nitta T, Sato Y, Nakanuma Y. GNAS and KRAS Mutations are Common in Intraductal Papillary Neoplasms of the Bile Duct. PLoS ONE. 2013;8(12):e81706. doi:10.1371/journal.pone.0081706.
29. Wu J, Jiao Y, Dal Molin M, Maitra A, de Wilde RF, Wood LD et al. Whole-exome sequencing of neoplastic cysts of the pancreas reveals recurrent mutations in components of ubiquitin-dependent pathways. Proc Natl Acad Sci U S A. 2011;108(52):21188-93. doi:1118046108 [pii]
10.1073/pnas.1118046108.
30. Alakus H, Babicky ML, Ghosh P, Yost S, Jepsen K, Dai Y et al. Genome-wide mutational landscape of mucinous carcinomatosis peritonei of appendiceal origin. Genome Med. 2014;6(5):43. doi:10.1186/gm559.
31. Nishikawa G, Sekine S, Ogawa R, Matsubara A, Mori T, Taniguchi H et al. Frequent GNAS mutations in low-grade appendiceal mucinous neoplasms. Br J Cancer. 2013;108(4):951-8. doi:10.1038/bjc.2013.47.

32. Matsubara A, Sekine S, Kushima R, Ogawa R, Taniguchi H, Tsuda H et al. Frequent GNAS and KRAS mutations in pyloric gland adenoma of the stomach and duodenum. J Pathol. 2013;229(4):579-87. doi:10.1002/path.4153.
33. Yamada M, Sekine S, Ogawa R, Taniguchi H, Kushima R, Tsuda H et al. Frequent activating GNAS mutations in villous adenoma of the colorectum. J Pathol. 2012;228(1):113-8. doi:10.1002/path.4012.
34. Jaiswal BS, Kljavin NM, Stawiski EW, Chan E, Parikh C, Durinck S et al. Oncogenic ERBB3 mutations in human cancers. Cancer Cell. 2013;23(5):603-17. doi:10.1016/j.ccr.2013.04.012.
35. Li M, Zhang Z, Li X, Ye J, Wu X, Tan Z et al. Whole-exome and targeted gene sequencing of gallbladder carcinoma identifies recurrent mutations in the ErbB pathway. Nat Genet. 2014;46(8):872-6. doi:10.1038/ng.3030.
36. Holbro T, Beerli RR, Maurer F, Koziczak M, Barbas CF, 3rd, Hynes NE. The ErbB2/ErbB3 heterodimer functions as an oncogenic unit: ErbB2 requires ErbB3 to drive breast tumor cell proliferation. Proc Natl Acad Sci U S A. 2003;100(15):8933-8. doi:10.1073/pnas.1537685100.
37. Jones S, Li M, Parsons DW, Zhang X, Wesseling J, Kristel P et al. Somatic mutations in the chromatin remodeling gene ARID1A occur in several tumor types. Hum Mutat. 2012;33(1):100-3. doi:10.1002/humu.21633.
38. Vogelstein B, Papadopoulos N, Velculescu VE, Zhou S, Diaz LA, Jr., Kinzler KW. Cancer genome landscapes. Science. 2013;339(6127):1546-58. doi:10.1126/science.1235122.
39. Davoli T, Xu AW, Mengwasser KE, Sack LM, Yoon JC, Park PJ et al. Cumulative haploinsufficiency and triplosensitivity drive aneuploidy patterns and shape the cancer genome. Cell. 2013;155(4):948-62. doi:10.1016/j.cell.2013.10.011.
40. Tamborero D, Gonzalez-Perez A, Perez-Llamas C, Deu-Pons J, Kandoth C, Reimand J et al. Comprehensive identification of mutational cancer driver genes across 12 tumor types. Sci Rep. 2013;3:2650. doi:10.1038/srep02650.
41. Brooks AN, Choi PS, de Waal L, Sharifnia T, Imielinski M, Saksena G et al. A pan-cancer analysis of transcriptome changes associated with somatic mutations in *U2AF1* reveals commonly altered splicing events. PLoS ONE. 2014;9(1):e87361. doi:10.1371/journal.pone.0087361.
42. Nakanishi Y, Seno H, Fukuoka A, Ueo T, Yamaga Y, Maruno T et al. Dclk1 distinguishes between tumor and normal stem cells in the intestine. Nat Genet. 2013;45(1):98-103. doi:http://www.nature.com/ng/journal/v45/n1/abs/ng.2481.html - supplementary-information.
43. Pickering CR, Zhou JH, Lee JJ, Drummond JA, Peng SA, Saade RE et al. Mutational landscape of aggressive cutaneous squamous cell carcinoma. Clin Cancer Res. 2014;20(24):6582-92. doi:10.1158/1078-0432.CCR-14-1768.
44. Merritt MA, Green AC, Nagle CM, Webb PM, Australian Cancer S, Australian Ovarian Cancer Study G. Talcum powder, chronic pelvic inflammation and NSAIDs in relation to risk of epithelial ovarian cancer. Int J Cancer. 2008;122(1):170-6. doi:10.1002/ijc.23017.
45. Bryan EJ, Watson RH, Davis M, Hitchcock A, Foulkes WD, Campbell IG. Localization of an ovarian cancer tumor suppressor gene to a 0.5-cM region between D22S284 and CYP2D, on chromosome 22q. Cancer Res. 1996;56(4):719-21.
46. Lee KR, Young RH. The distinction between primary and metastatic mucinous carcinomas of the ovary: gross and histologic findings in 50 cases. Am J Surg Pathol. 2003;27(3):281-92.
47. Thompson ER, Doyle MA, Ryland GL, Rowley SM, Choong DY, Tothill RW et al. Exome sequencing identifies rare deleterious mutations in DNA repair genes FANCC and BLM as potential breast cancer susceptibility alleles. PLoS Genet. 2012;8(9):e1002894. doi:10.1371/journal.pgen.1002894.

48. Gonzalez-Perez A, Perez-Llamas C, Deu-Pons J, Tamborero D, Schroeder MP, Jene-Sanz A et al. IntOGen-mutations identifies cancer drivers across tumor types. Nat Methods. 2013;10(11):1081-2. doi:10.1038/nmeth.2642.
49. Wolff AC, Hammond ME, Schwartz JN, Hagerty KL, Allred DC, Cote RJ et al. American Society of Clinical Oncology/College of American Pathologists guideline recommendations for human epidermal growth factor receptor 2 testing in breast cancer. J Clin Oncol. 2007;25(1):118-45. doi:JCO.2006.09.2775 [pii]
10.1200/JCO.2006.09.2775.

Table 1. Cohort summary

Clinical feature	Discovery (n=24)	Validation (n=58)
Type:		
Benign	5	17
Borderline	8	21
Carcinoma	11	20
Grade (carcinoma)		
1	5	6
2	3	10
3	2	4
Not known	1	
Stage (carcinoma)		
1	7	16
2	1	1
3	1	1
Not known	2	2
Age (average ± standard deviation)		
Benign	49.8 ± 5.9	59.5 ± 11.3
Borderline	55.4 ± 15.8	53.6 ± 16.0
Carcinoma	53.8 ± 11.0	53.8 ± 11.1
Laterality		
Unilateral	21	4
Bilateral	2	54
Not known	1	
Size		
<10 cm	2	1
≥10 cm	21	57
Not known	1	

Table 2: Candidate driver genes with significantly recurrent somatic mutations in mucinous ovarian tumors.

Gene	Exome cohort			SMG prediction				Validation cohort			Overall
					MuSiC *q*-value						
	Mutated samples	Nonsense, frameshift indel, splice	Inframe indel, missense	OncodriveFM *q*-value	FCPT	LRT	CT	Mutated samples	Nonsense, frameshift indel, splice	Inframe indel, missense	Mutated samples
KRAS	12	0	12	1.34×10^{-13}	0	0	0	32	0	33	44/82
TP53	7	1	6	3.66×10^{-11}	2.86×10^{-7}	1.41×10^{-9}	1.17×10^{-12}	15	0	15	22/82
BRAF	6	0	6	2.45×10^{-8}	7.77×10^{-7}	0	6.18×10^{-12}	4	0	5	10/82
CDKN2A	5	5	2	0.0043	1.20×10^{-10}	0	7.93×10^{-17}	5	5	0	10/63
RNF43	5	5	0	4.65×10^{-6}	-	0.0009	0.0004	3	2	1	8/65[b]
ELF3	3	2	1	0.0079	-	0.0003	0.0004	1	1	0	4/65
ARID1A	2	2	0	0.0164	-	0.0933	-	-	-	-	2/24
DCLK1	2	0	2	-	-	0.0569	-	-	-	-	2/24
ERBB3	2	0	3	-	-	0.0014	0.0374	0	0	0	2/43
FBXW7	2	1	1	0.0207	-	-	-	-	-	-	2/24
GNAS[a]	2	0	2	9.05×10^{-8}	-	-	-	3	0	3	5/81
KLF5	2	2	0	0.0164	-	0.0056	0.0536	0	0	0	2/43
LPHN3	2	0	2	0.0493	-	-	-	-	-	-	2/24
LRRK2	2	1	1	0.0997	-	-	-	-	-	-	2/24
TTF1	2	0	2	-	-	0.0569	-	-	-	-	2/24

All non-synonymous mutations in listed genes were validated by Sanger sequencing. CT, convolution test; FCPT, Fisher's combined *P*-value test; LRT, likelihood ratio test; SMG, significantly mutated gene. [a]Only mutations involving the hotspot codon 201 are reported. [b]Includes samples from Ryland *et al.*[9] plus 16 additional samples.

Figure legends

Figure 1: Mutational landscape of MOTs identified by exome sequencing. Samples are grouped according to pathological classification and ordered from lowest to highest mutation frequency. (A) Somatic mutation frequency (left Y-axis) and number of coding mutations by consequence (right Y-axis). (B) Relative frequency of somatic mutations according to base substitution type. Substitutions were categorized by the six possible base-pair changes.

Figure 2: Candidate driver genes in MOTs. Significantly mutated genes identified by OncodriveFM and MuSiC analyses are arranges vertically by their frequency of mutated samples in the whole exome sequencing data. Color indicates mutation consequence. Selected genes were also investigated in a validation cohort of mucinous tumors. Each column denotes an individual tumor (ordered as listed in Additional File 1, Table S1), which have been arranged to emphasize mutational groups. Genomic aberrations in other MAPK pathway genes were also screened for mutations. LOH, loss of heterozygosity.

Figure 3: Distribution of somatic mutations identified in novel significantly mutated genes. *ELF3*, *KLF5*, *GNAS* and *ERBB3* are shown in the context of protein domains as predicted by UniProt, with somatic mutations identified in the exome (closed circle) and validation (open circle) cohorts mapped to each gene. PNT, pointed domain; TAD, transactivation domain; SAR, serine- and aspartic acid-rich domain; AT hook & NLS, AT-hook domain and nuclear localization signal; ETS, DNA binding domain; C2H2, zinc-finger C2H2 domain; GTP, GTP nucleotide binding region; I-IV, extracellular domains I, II, III and IV; TKD, tyrosine kinase domain.

Figure 1
Click here to download Figure: Ryland_MOTexome_Figure1.tif

Figure 1

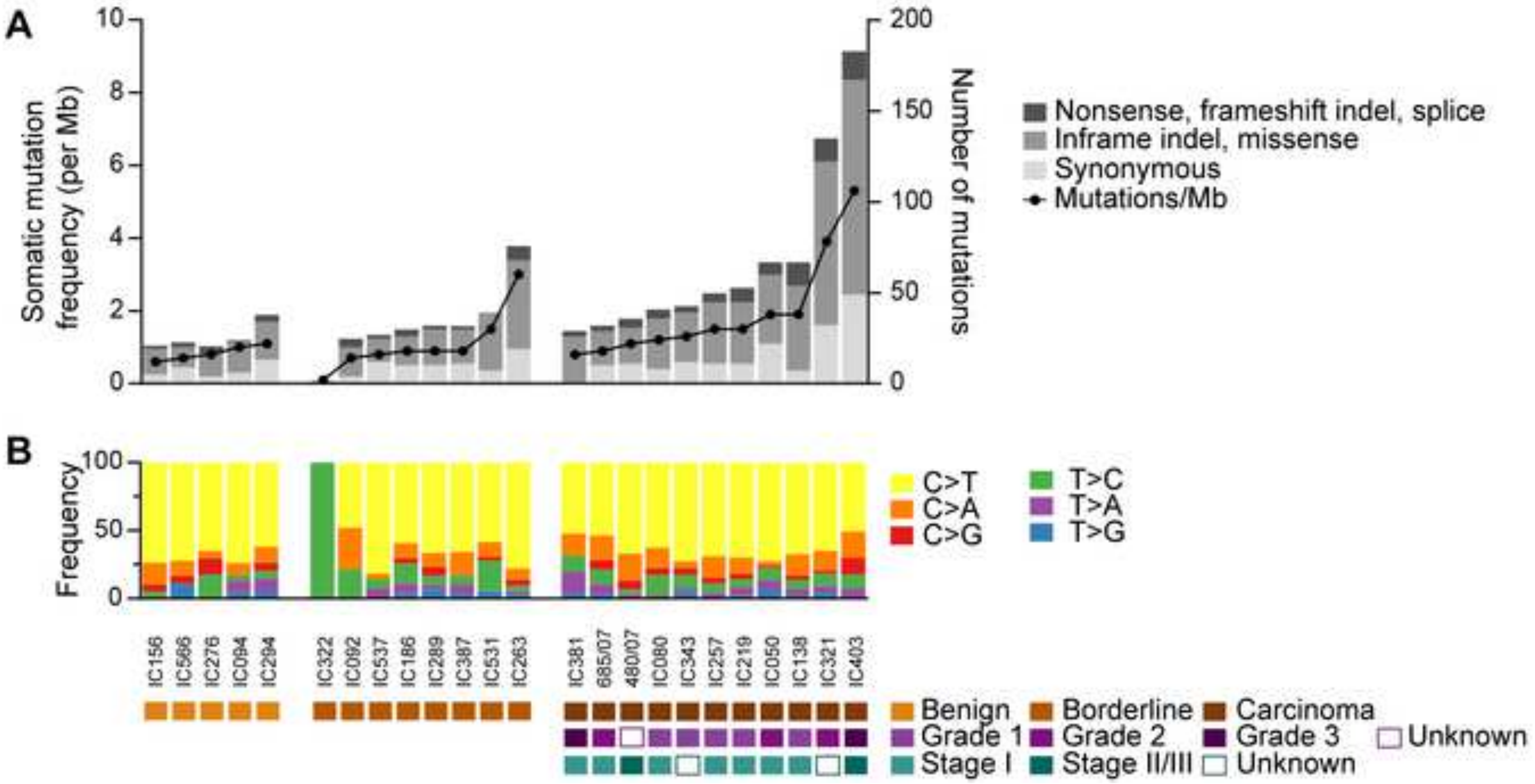

Figure 2
Click here to download Figure: Ryland_MOTexome_Figure2.tif

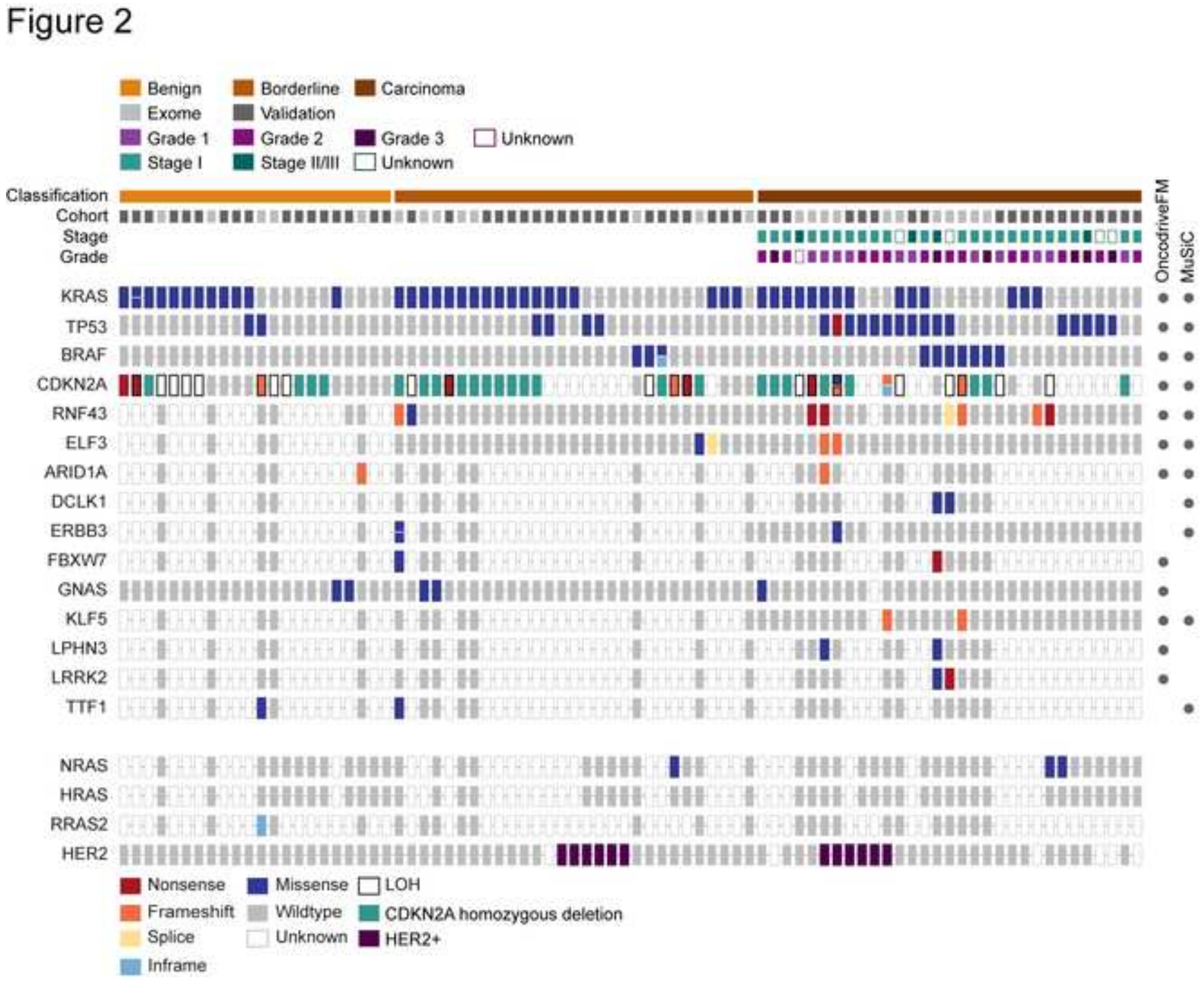

Figure 3
Click here to download Figure: Ryland_MOTexome_Figure3.tif

Figure 3

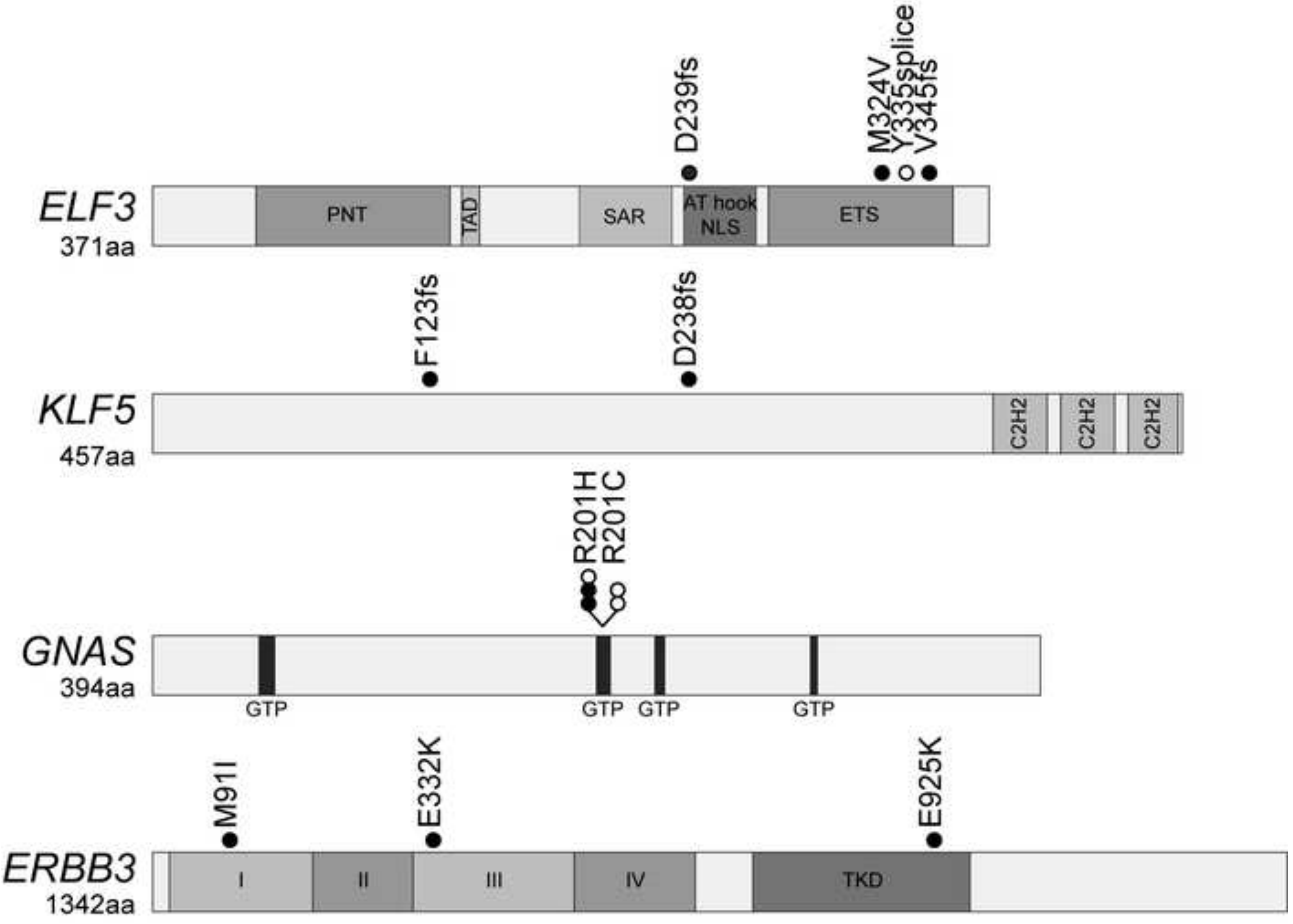

Supplementary Tables S1-S6

Click here to download Supplementary Material: Additional File 1 - Gorringe corr.xlsx

Supplementary Figures S1-S5

Click here to download Supplementary Material: Additional File 2.pdf
