# Supplementary material for: Erratum to: Mutational landscape of mucinous ovarian carcinoma and its neoplastic precursors
Source: Genome Med. 2017 Jan 12;9:1. doi: 10.1186/s13073-016-0392-y (PMC5228100; doi:10.1186/s13073-016-0392-y)
Supplement: Additional file 2: Figure S1. — Nucleotide substitution frequency and context. Figure S2. RRAS2 somatic mutation. Figure S3. Genetic comparison between mucinous ovarian tumors and mucinous cancers from other anatomical sites. Figure S4. ELF3 somatic mutations. Figure S5. H&E stained sections of frozen tissues used for exome discovery cohort. (PDF 9813 kb) [file 13073_2016_392_MOESM1_ESM.pdf]

Supplementary figures

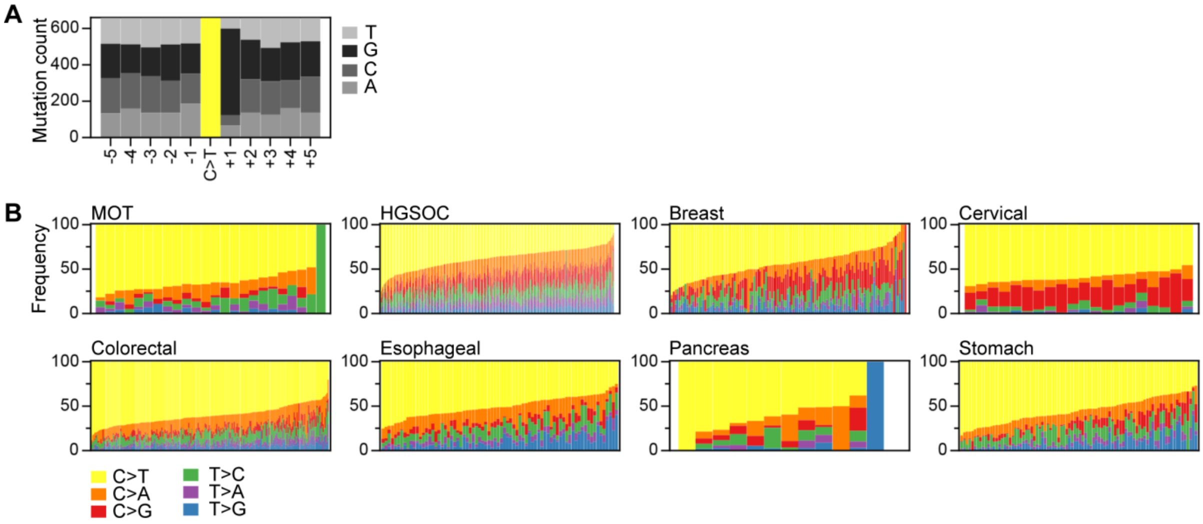

Supplementary Figure S1: (A) Nucleotide frequency of the five base pairs preceding and following C>T/G>A transitions in MOTs, demonstrating a predominance of the NpCpG trinucleotide signature. (B) Relative frequency of somatic mutations according to the six possible base-pair substitutions in mucinous ovarian tumors (MOT) from this study and high-grade serous ovarian carcinomas (HGSOC), breast, cervical, colorectal, esophageal, pancreatic and gastric cancers derived from Lawrence *et al*. (14). Samples are ordered according to C>T/G>A mutation frequency.

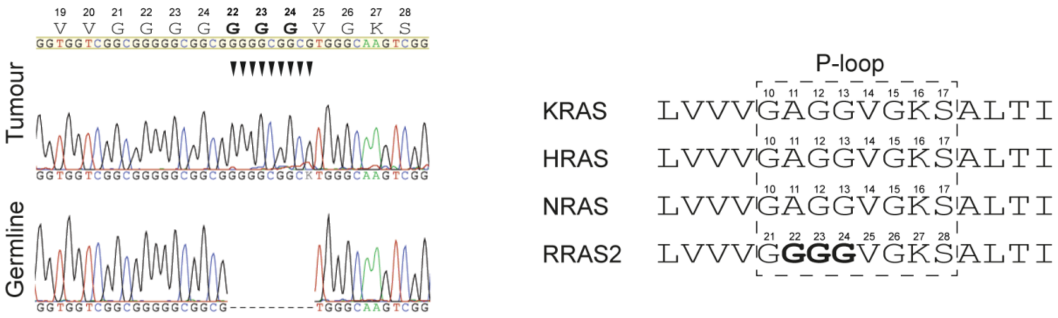

Supplementary Figure S2: *RRAS2* somatic mutation. The p.Gly22_Gly24dup *RRAS2* mutation was validated by Sanger sequencing. Mutated positions are indicated by the arrows. Homozygous expression of the mutant allele was observed. This three amino acid reiteration occurs within the P-loop region of ras proteins and corresponding to residues 11-13 of *KRAS*, *HRAS* and *NRAS*, as depicted in the sequence alignment. Codon numbering is shown above the amino acid sequence. Mutated positions are indicated in bold.

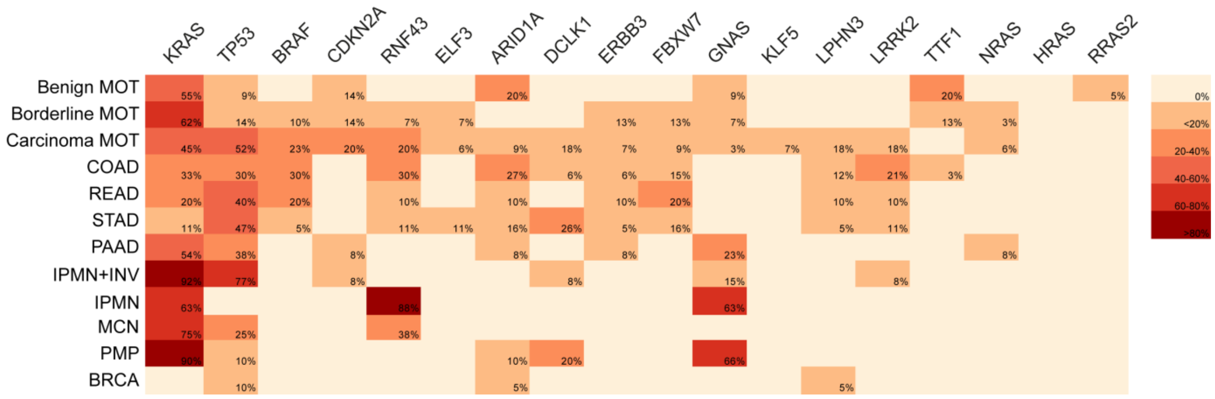

Supplementary Figure S3: Genetic comparison between mucinous ovarian tumors and mucinous cancers from other anatomical sites. The frequency of known and candidate mucinous ovarian tumor (MOT) genes identified in this study is shown for tumors with a mucinous histology including breast carcinoma (BRCA, n=21; data from TCGA), colon adenocarcinoma (COAD, n=33; TCGA), rectum adenocarcinoma (READ, n=10; TCGA), stomach adenocarcinoma (STAD, n=19; TCGA), pseudomyxoma peritonei of appendiceal origin from Alakus *et al*. (36) (PMP, n=29 for *KRAS*/*GNAS* and n=10 for all other genes), and a spectrum of pancreatic mucinous tumors including mucinous carcinoma (PAAD, n=13; TCGA and ICGC), intraductal papillary mucinous neoplasm with invasion (IPMN+INV, n=13; ICGC), intraductal papillary mucinous neoplasm (IPMN, n=8) and mucinous cystic neoplasm (MCN, n=8) from Wu *et al*. (35).

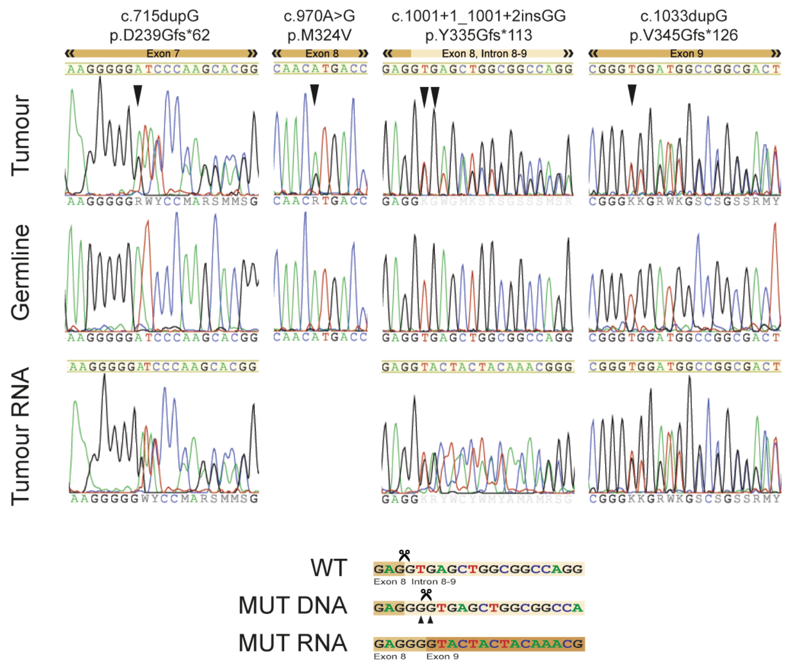

Supplementary Figure S4: *ELF3* somatic mutations. (TOP) Three *ELF3* mutations identified by exome sequencing (p.D239Gfs*62, p.M324V and p.V345Gfs*126) were validated in the tumor and matched germline by Sanger sequencing. An additional splice site mutation (c.1001+1_1001+2insGG) was identified in the validation screen. Arrows indicate the mutation locations. (BOTTOM) Expression of the mutant allele was investigated by cDNA sequencing in the three tumor samples that had RNA available (extracted using the RNeasy Kit, Qiagen). Total RNA (200 ng) was converted to cDNA using the SuperScript VILO cDNA Synthesis Kit (Invitrogen) and assessed by end-point PCR using HotStar Taq polymerase (Qiagen) and Sanger sequencing as described in the Methods and Supplementary Table S4. The mutant allele was expressed consistent with the heterozygous variant allele frequency in the exome data. In addition, cDNA sequencing confirmed the functional nature of the splice site mutation. MUT, mutant; WT, wildtype.

Supplementary Figure S5 H&E stained sections of frozen tissues used for exome discovery cohort

Benign

IC094

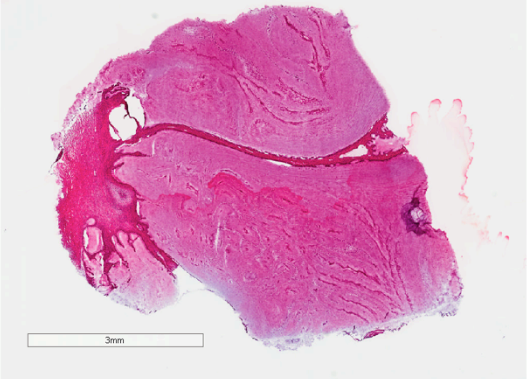

IC566

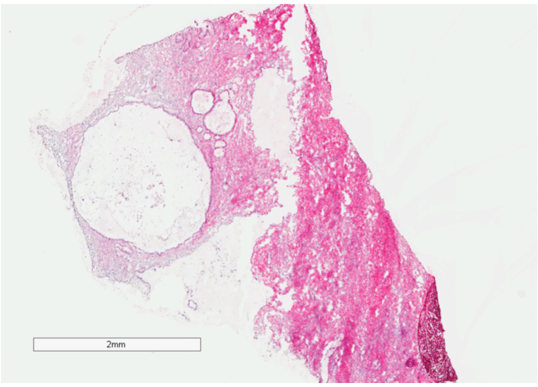

IC276

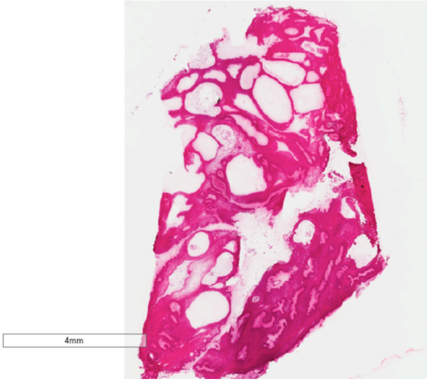

IC294

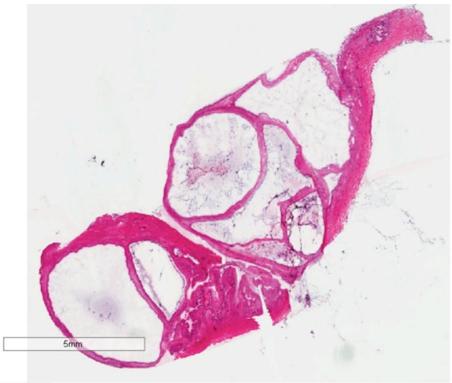

IC156

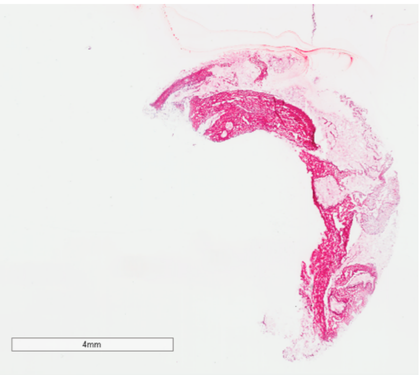

Borderline

IC092

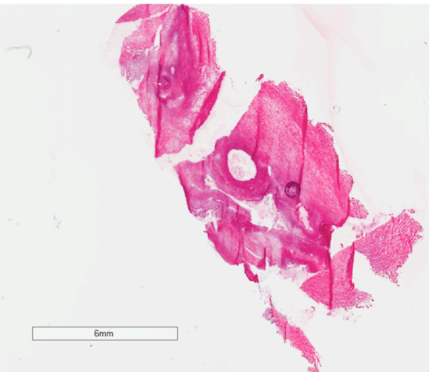

IC186

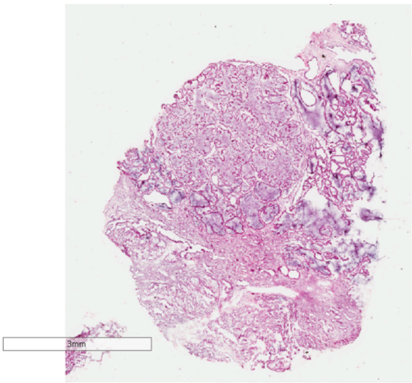

IC263

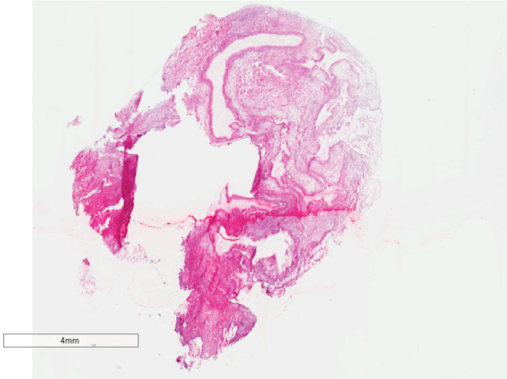

IC289

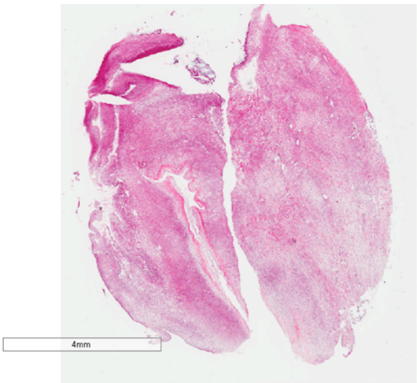

Borderline cont.

IC322

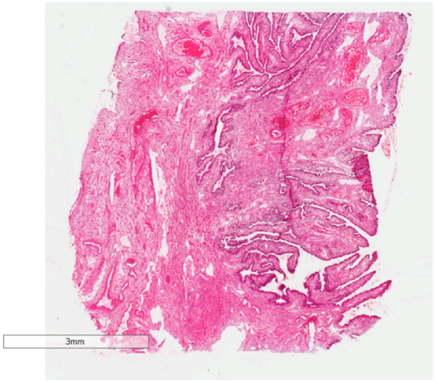

IC387

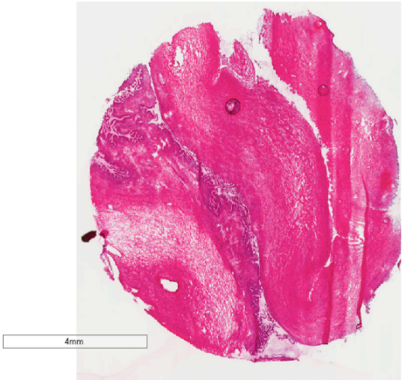

IC531

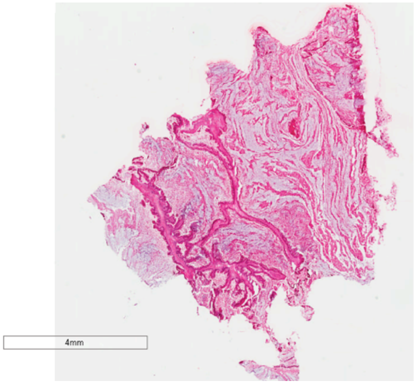

IC537

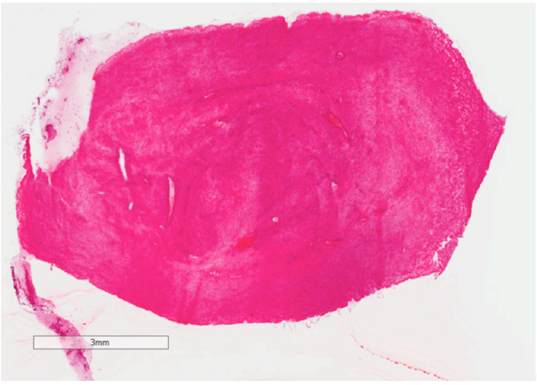

Carcinoma

IC050

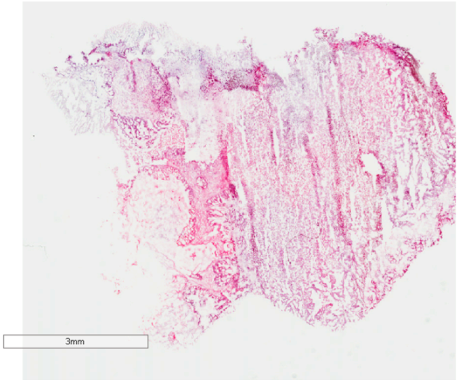

IC080

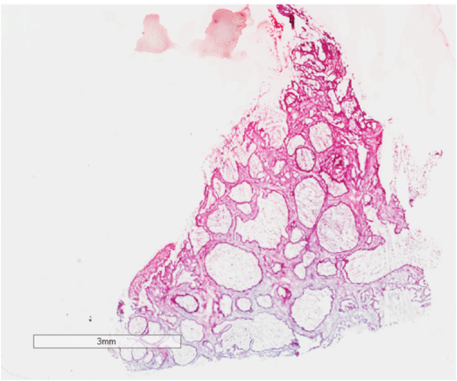

IC138

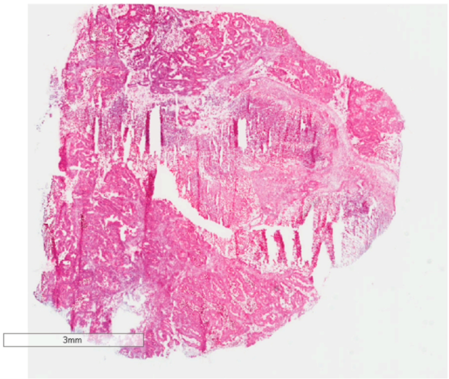

IC257

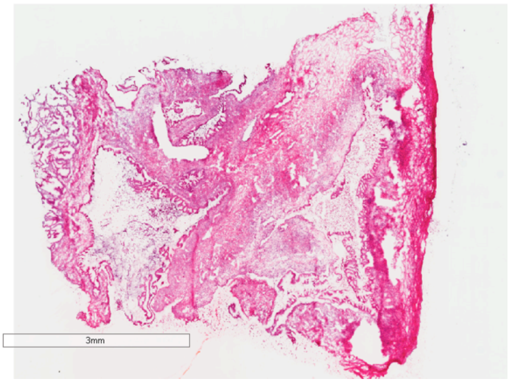

Carcinoma cont.

IC219

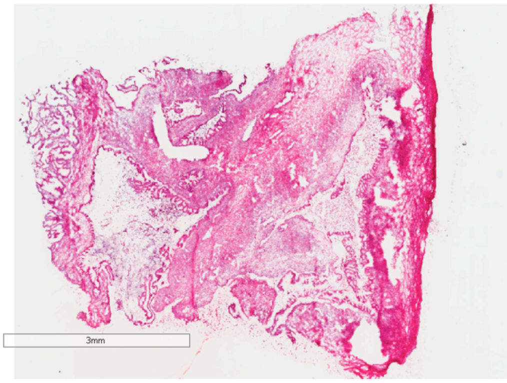

IC321

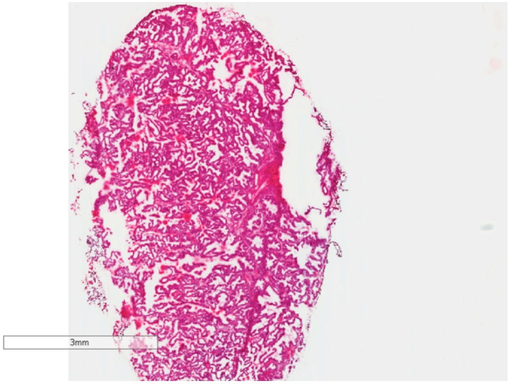

IC343

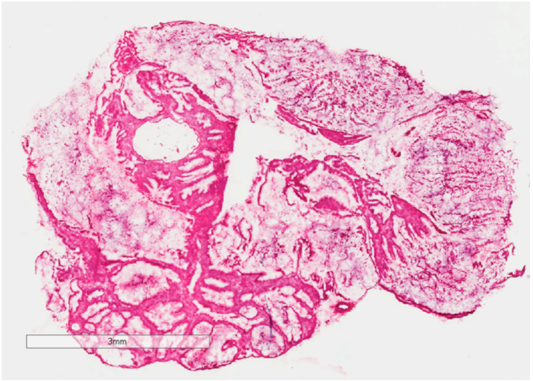

IC381

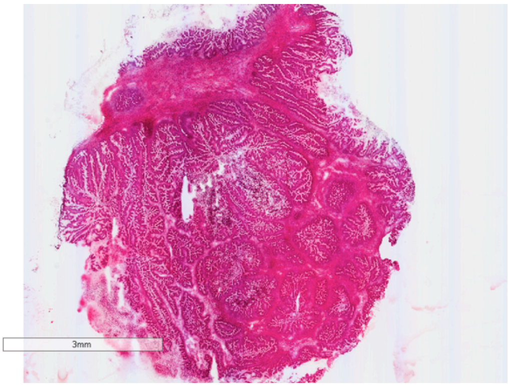

IC403

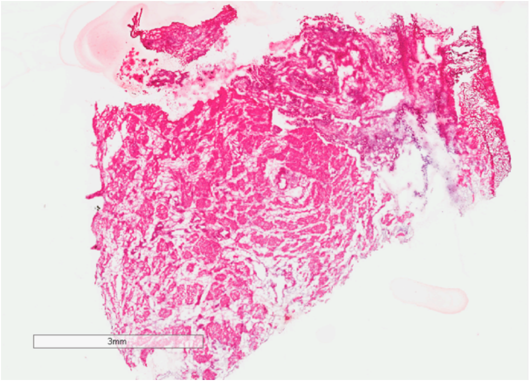

480/07

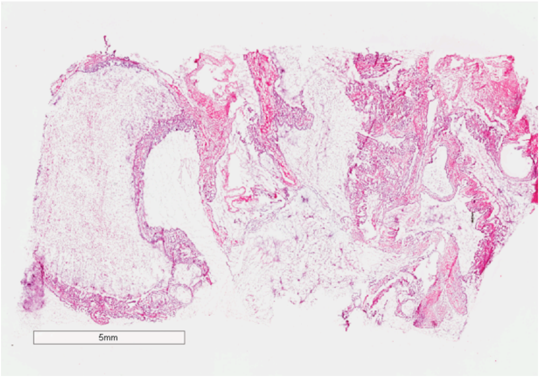

685/07

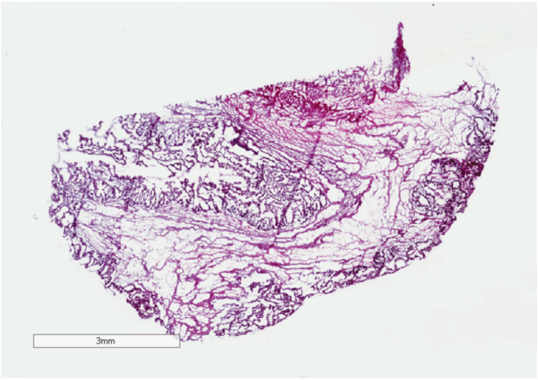

Supplementary References

(Note: reference numbers refer those used in the main article)

14. Lawrence MS, Stojanov P, Polak P, Kryukov GV, Cibulskis K, Sivachenko A, et al. Mutational heterogeneity in cancer and the search for new cancer-associated genes. Nature. 2013;499:214-8.

36. Alakus H, Babicky ML, Ghosh P, Yost S, Jepsen K, Dai Y, et al. Genome-wide mutational landscape of mucinous carcinomatosis peritonei of appendiceal origin. Genome Med. 2014;6:43.

35. Wu J, Jiao Y, Dal Molin M, Maitra A, de Wilde RF, Wood LD, et al. Whole-exome sequencing of neoplastic cysts of the pancreas reveals recurrent mutations in components of ubiquitin-dependent pathways. Proc Natl Acad Sci U S A. 2011;108:21188-93.
